# Supplementary material for: Chemical Structure Diversity and Extensive Biological Functions of Specialized Metabolites in Rice
Source: Int J Mol Sci. 2023 Dec 2;24(23):17053. doi: 10.3390/ijms242317053 (PMC10707428; doi:10.3390/ijms242317053)
Supplement: Supplementary file 1 [file ijms-24-17053-s001.zip › ijms-2713560-supplementary.pdf]

International Journal of *Molecular Sciences*

MDPI

Review

Chemical Structure Diversity and Extensive Biological Functions of Specialized Metabolites in Rice

Huiwen Zhou †, Jinjin Zhang †, Liping Bai, Jiayi Liu, Hongdi Li, Juan Hua * and Shihong Luo *

Appendix A

Figure legends:

Figure S1. Chemical structures of rice monoterpenoids and sesquiterpenoids (**1–49**).

Figure S2. Chemical structures of rice diterpenoids (**50–101**).

Figure S3. Chemical structures of rice triterpenoids (**102–136**).

Figure S4. Chemical structures of rice steroids (**137–181**).

Figure S5. Chemical structures of rice phenolic compounds (**182–372**).

Figure S6. Chemical structures of rice alkaloids (**373–413**).

Figure S7. Chemical structures of other types of rice compounds (**414–439**).

Table S1. Terpenoids and steroids identified from rice (**1–181**).

Table S2. Phenolic compounds identified from rice (**182–372**).

Table S3. Alkaloids identified from rice (**373–413**).

Table S4. Other types of compounds identified from rice (**414–439**).

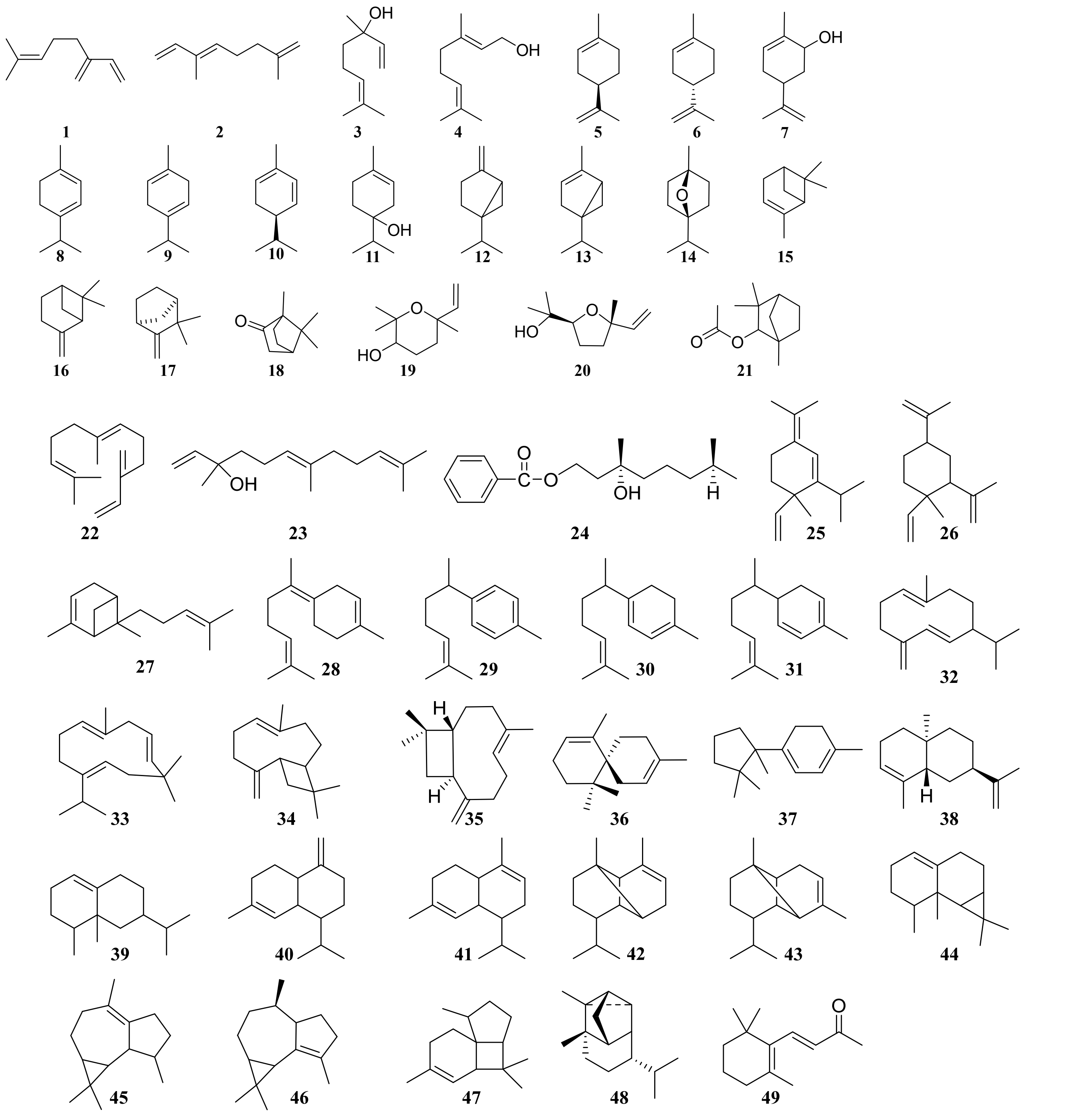

Figure S1. Chemical structures of rice monoterpenoids and sesquiterpenoids (**1–49**).

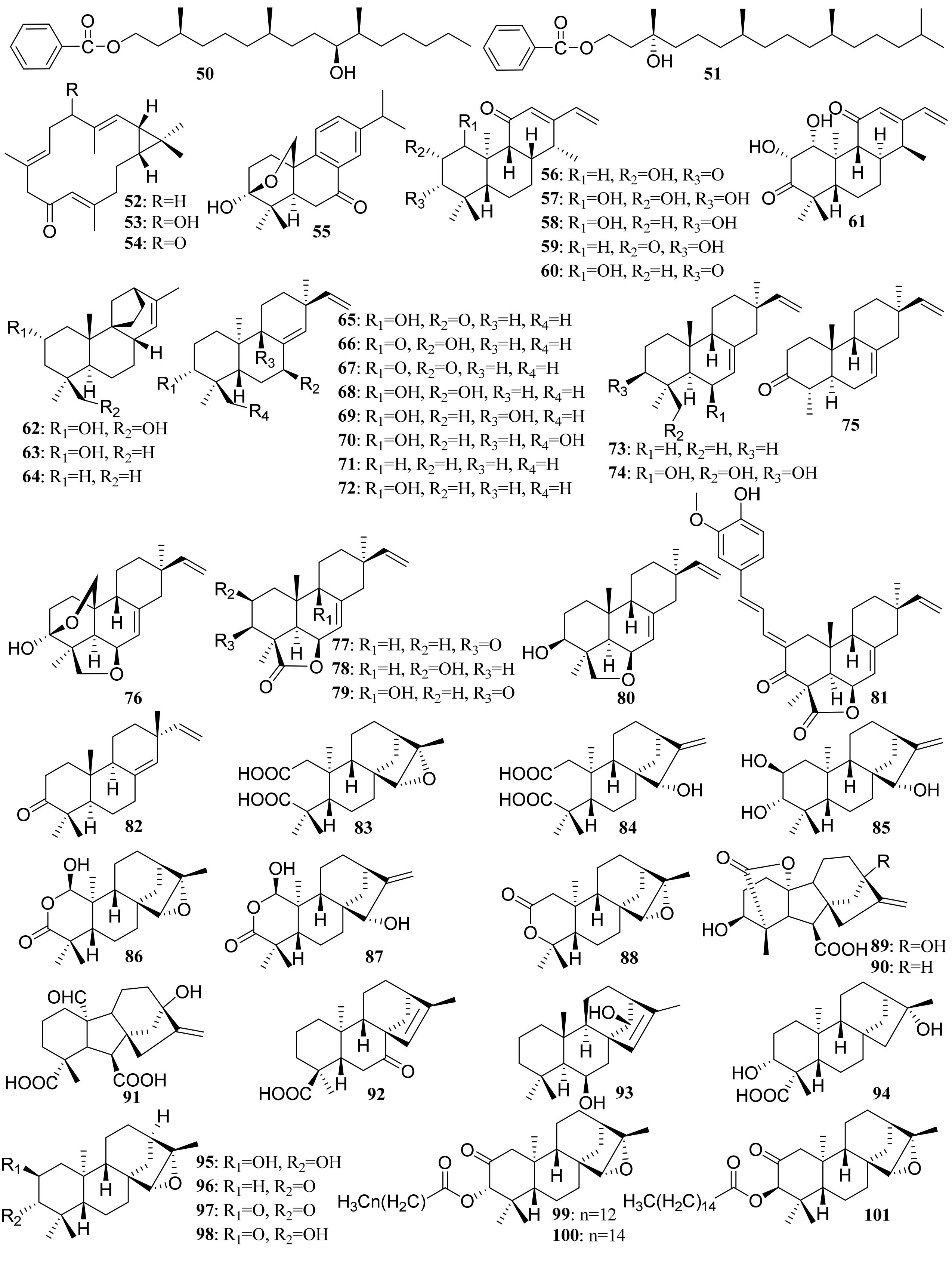

Figure S2. Chemical structures of rice diterpenoids (**50–101**).

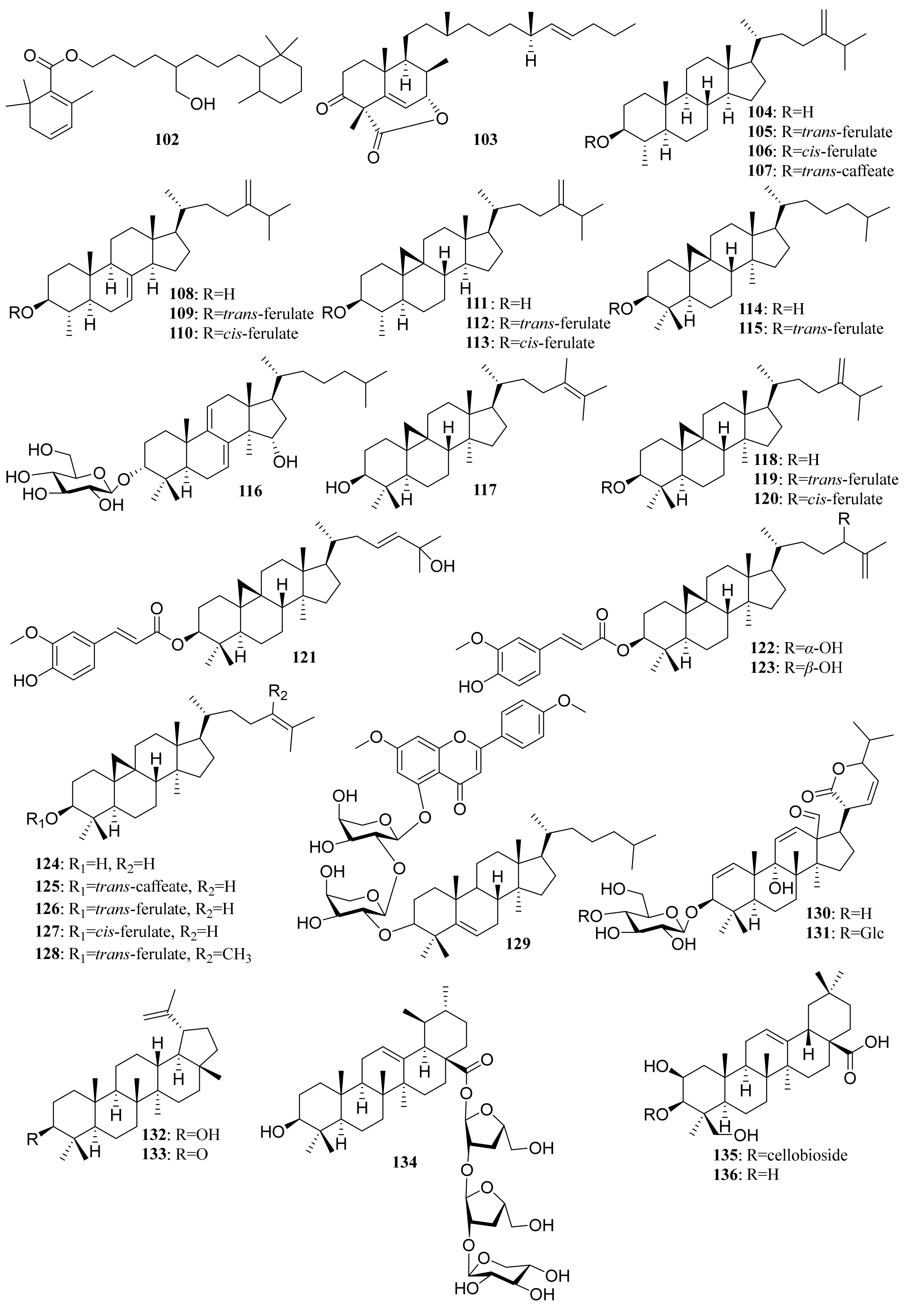

Figure S3. Chemical structures of rice triterpenoids (**102–136**).

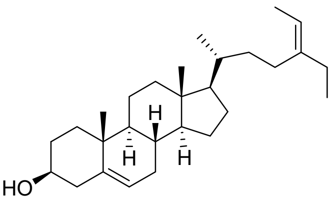

137

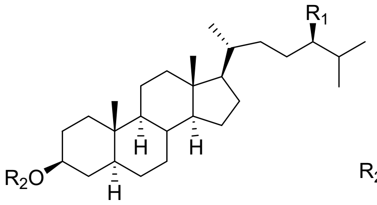

138: R_1=CH_2CH_3, R_2=H
139: R_1=CH_3, R_2=H
140: R_1=CH_3, R_2=*trans*-ferulate
141: R_1=CH_2CH_3, R_2=*trans*-ferulate
142: R_1=CH_2CH_3, R_2=*cis*-ferulate

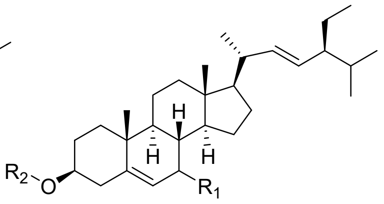

143: R_1=H, R_2=H
144: R_1=O, R_2=H
145: R_1=H, R_2=*trans*-ferulate
146: R_1=H, R_2=*cis*-ferulate

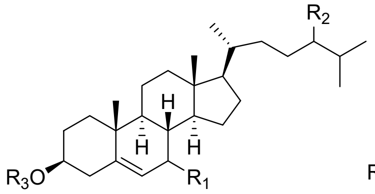

147: R_1=H, R_2=H, R_3=H
148: R_1=H, R_2= =CH_2, R_3=*cis*-ferulate
149: R_1=H, R_2=CH_3, R_3=*cis*-ferulate
150: R_1=H, R_2= =CH_2, R_3=*trans*-ferulate
151: R_1=H, R_2=α-CH_3, R_3=H
152: R_1=H, R_2= =CH_2, R_3=H
153: R_1=H, R_2= =$CHCH_3$, R_3=H
154: R_1=H, R_2=β-CH_2CH_3, R_3=H
155: R_1=α-OH, R_2=β-CH_2CH_3, R_3=H
156: R_1=β-OH, R_2=β-CH_2CH_3, R_3=H
157: R_1=α-OCH_3, R_2=β-CH_2CH_3, R_3=H
158: R_1=H, R_2=α-CH_3, R_3=*trans*-caffeate
159: R_1=H, R_2=α-CH_3, R_3=*trans*-ferulate
160: R_1=H, R_2=H, R_3=*trans*-ferulate
161: R_1=H, R_2= =CH_2, R_3=*trans*-ferulate
162: R_1=H, R_2=β-CH_2CH_3, R_3=trans-ferulate
163: R_1=H, R_2=β-CH_2CH_3, R_3=*cis*-ferulate
164: R_1=H, R_2=β-CH_2CH_3, R_3=Glc
165: R_1=H, R_2=β-CH_2CH_3, R_3=(3'-1)-D-Glc-(4-1)-D-Glc
166: R_1=H, R_2=β-CH_2CH_3, R_3=(3'-1)-D-Glc-(3-1)-D-Glc
167: R_1=H, R_2=β-CH_2CH_3, R_3=(3'-1)-D-Glc-(4-1)-D-Glc-(4-1)-D-Glc
168: R_1=H, R_2=β-CH_2CH_3, R_3=(3'-1)-D-Glc-(4-1)-D-Glc-(4-1)-D-Glc-(4-1)-D-Glc
169: R_1=H, R_2=β-CH_2CH_3, R_3=(3'-1)-D-Glc-(4-1)-D-Glc-(4-1)-D-Glc-(4-1)-D-Glc-(4-1)-D-Glc

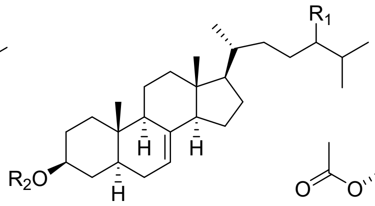

170: R_1= =CH_2, R_2=H
171: R_1= =$CHCH_3$, R_2=H
172: R_1=β-CH_3, R_2=H
173: R_1=β-CH_2CH_3, R_2=H
174: R_1=α-CH_3, R_2=*trans*-ferulate
175: R_1=β-CH_2CH_3, R_2=*trans*-ferulate

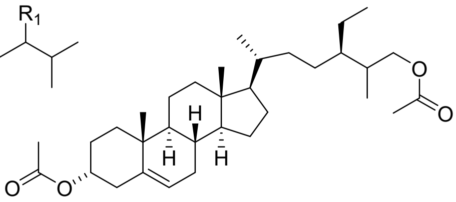

176

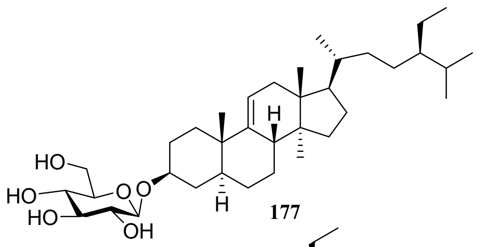

177

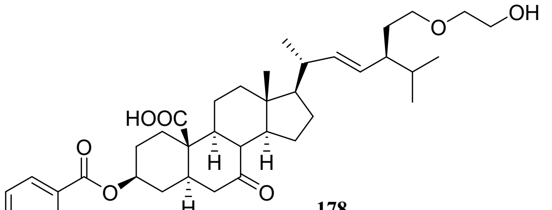

178

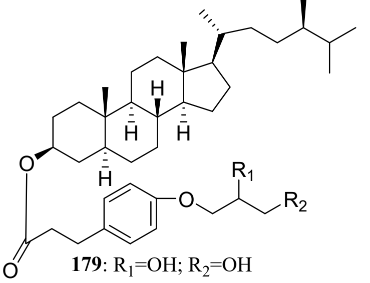

179: R_1=OH; R_2=OH
180: R_1=H; R_2=CH_3

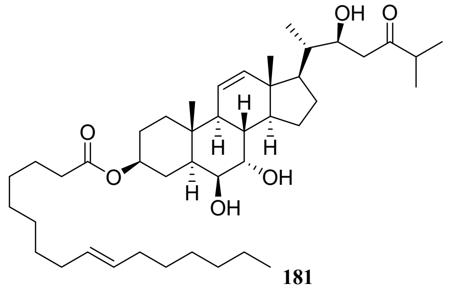

181

Figure S4. Chemical structures of rice steroids (**137–181**).

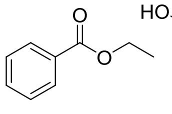

182

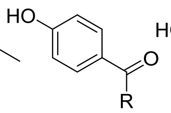

183: R=H
184: R=OH

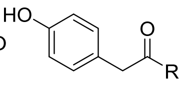

185: R=H
186: R=OH

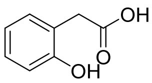

187

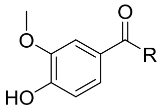

188: R=H
189: R=OH

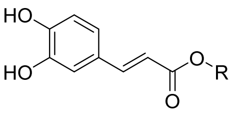

190: R=H
191: $R=CH_3$

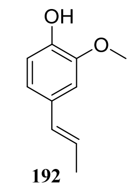

192

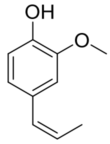

193

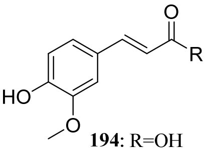

194: R=OH
195: $R=OCH_3$
196: R=H

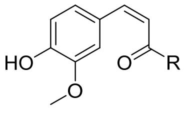

197: R=OH
198: $R=OCH_3$

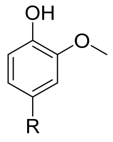

199: R=H
200: $R=CH{=}CHCH_3$ *trans*
201: $R=CH_2CH_2CH_3$
202: $R=CH_2COCH_3$

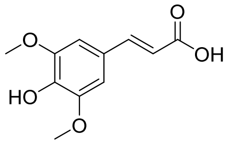

203

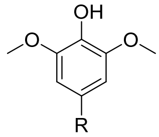

204: R=H
205: $R=CH_3$
206: $R=CH_2CH_3$
207: $R= CH_2CH{=}CH_2$
208: $R=CH{=}CH_2$
209: $R=CH{=}CHCH_3$
210: R= CHO
211: R=COOH
212: $R=COCH_3$

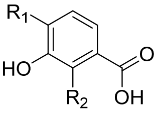

213: R_1=H, R_2=H
214: R_1=OH, R_2=H
215: R_1=OH, R_2=OH

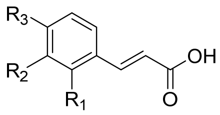

216: R_1=H, R_2=H, R_3=H
217: R_1=OH, R_2=H, R_3=H
218: R_1=H, R_2=OH, R_3=H
219: R_1=H, R_2=H, R_3=OH

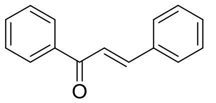

220

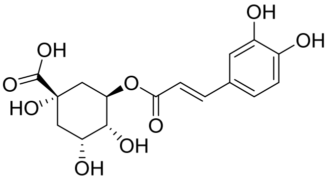

221

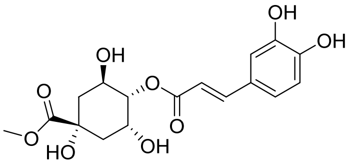

222

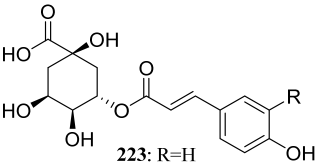

223: R=H
224: $R=OCH_3$

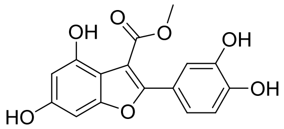

225

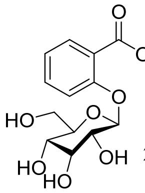

226

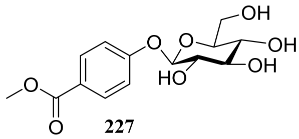

227

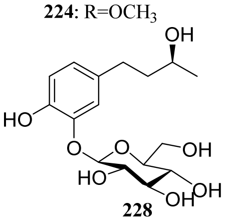

228

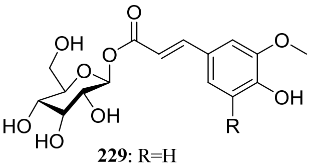

229: R=H
230: $R=OCH_3$

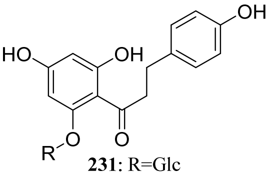

231: R=Glc
232: R=H

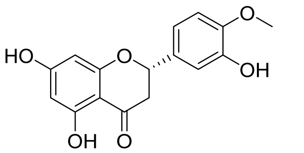

233

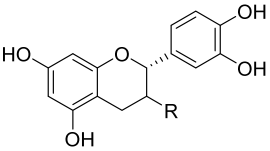

234: R=α-OH
235: R=β-OH

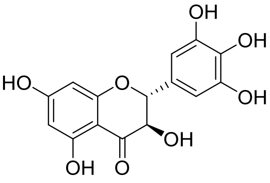

236

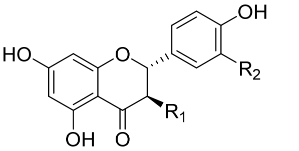

237: R_1=H, R_2=OH
238: R_1=OH, R_2=OH
239: R_1=OH, R_2=H

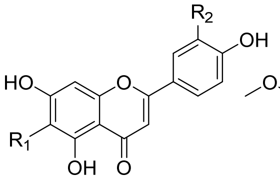

240: R_1=H, R_2=OH
241: $R_1=OCH_3$, R_2=H
242: R_1=H, R_2=H

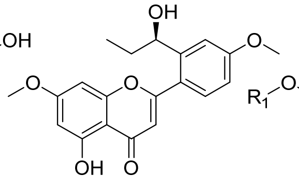

243

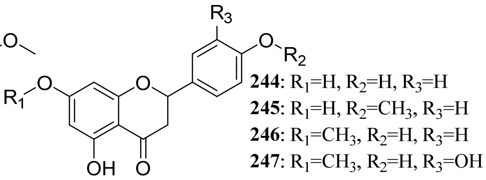

244: R_1=H, R_2=H, R_3=H
245: R_1=H, $R_2=CH_3$, R_3=H
246: $R_1=CH_3$, R_2=H, R_3=H
247: $R_1=CH_3$, R_2=H, R_3=OH
248: $R_1=CH_3$, R_2=Xyl, R_3=H
249: R_1=Xyl, R_2=H, R_3=H
250: R_1=H, $R_2=CH_3$, R_3=OH
251: R_1=Rut, R_2=H, R_3=H
252: R_1=Rha-Glc, $R_2=CH_3$, R_3=OH
253: R_1=Glc, R_2=H, R_3=OH
Xyl=Xylopyranoside

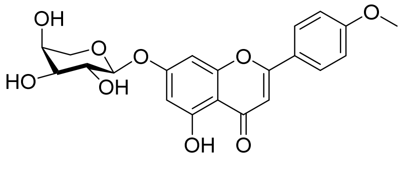

254

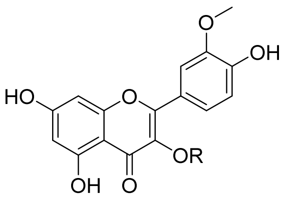

255: R=Rha
256: R=Rut

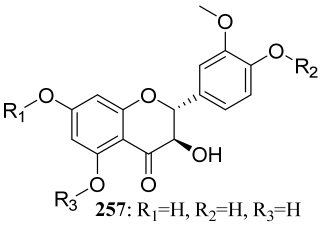

257: R_1=H, R_2=H, R_3=H
258: R_1=Glc, R_2=H, R_3=H
259: R_1=H, R_2=Glc, R_3=H
260: R_1=H, R_2=H, R_3=Glc

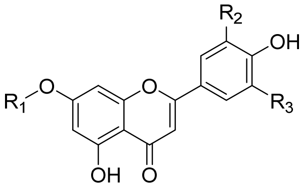

261: R_1=H, R_2=OH, R_3=OH
262: R_1=Rut, R_2=H, R_3=H
263: R_1=Rut, R_2=OCH_3, R_3=H
264: R_1=Glc, R_2=Glc, R_3=H
265: R_1=Glc, R_2=OH, R_3=H
266: R_1=Glc, R_2=H, R_3=H
267: R_1=Rut, R_2=OH, R_3=H

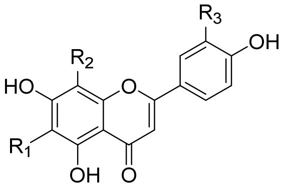

268: R_1=Glc, R_2=H, R_3=OH
269: R_1=Glc, R_2=H, R_3=H
270: R_1=Glc, R_2=H, R_3=OCH_3
271: R_1=α-L-Ara, R_2=β-L-Ara, R_3=H
272: R_1=α-L-Ara, R_2=β-L-Ara, R_3=OCH_3
273: R_1=H, R_2=Glc-2''-O-Glc, R_3=H
274: R_1=Glc(1''-2) α-L-Ara, R_2=H, R_3=OH
275: R_1=β-D-Glc, R_2=β-L-Ara, R_3=H
276: R_1=β-D-Glc, R_2=α-L-Ara, R_3=H
277: R_1=H, R_2=Glc, R_3=H

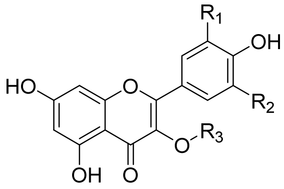

278: R_1=H, R_2=H, R_3=H
279: R_1=H, R_2=H, R_3=Rut
280: R_1=H, R_2=H, R_3=Rob
281: R_1=OH, R_2=OH, R_3=H
282: R_1=OH, R_2=H, R_3=H
283: R_1=OH, R_2=H, R_3=Glc
284: R_1=OH, R_2=H, R_3=Gal
285: R_1=OH, R_2=H, R_3=Rha-Glc
286: R_1=OCH_3, R_2=OCH_3, R_3=Rut
287: R_1=OCH_3, R_2=OCH_3, R_3=Glc
288: R_1=H, R_2=H, R_3=Glc
Rob=Robinobioside

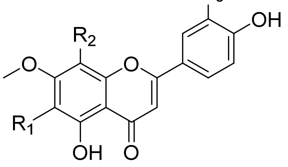

289: R_1=Glc, R_2=H, R_3=OCH_3
290: R_1=Glc, R_2=H, R_3=H

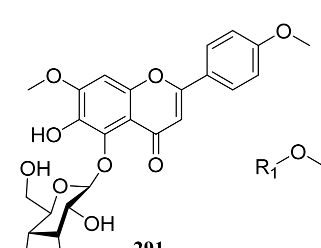

291

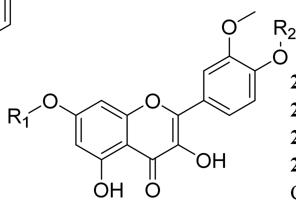

292: R_1=H, R_2=H
293: R_1=Glc, R_2=Glc
294: R_1=H, R_2=Glc
295: R_1=Cell, R_2=H
Cell=β-D-Cellobiosyl

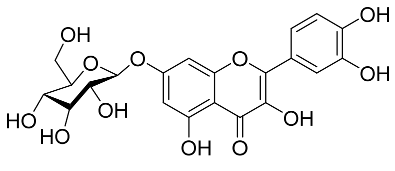

296

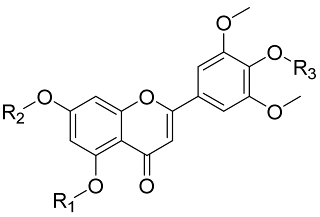

297: R_1=H, R_2=H, R_3=H
298: R_1=H, R_2=Glc, R_3=H
299: R_1=Glc, R_2=H, R_3=H
300: R_1=H, R_2=Rut, R_3=H
301: R_1=H, R_2=Neo, R_3=H
302: R_1=H, R_2=Glc(1''-2)GluA, R_3=H
303: R_1=H, R_2=H, R_3=Glc

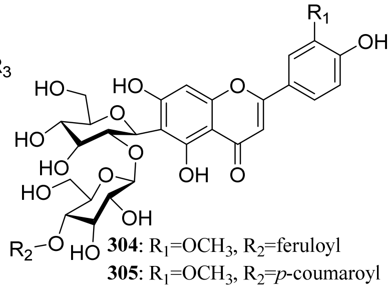

304: R_1=OCH_3, R_2=feruloyl
305: R_1=OCH_3, R_2=*p*-coumaroyl
306: R_1=OCH_3, R_2=H
307: R_1=H, R_2=feruloyl
308: R_1=H, R_2=*p*-coumaroyl
309: R_1=OMe, R_2=H

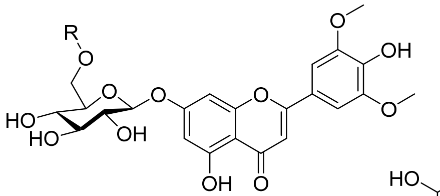

310: R=Malonyl
311: R=Sinapol

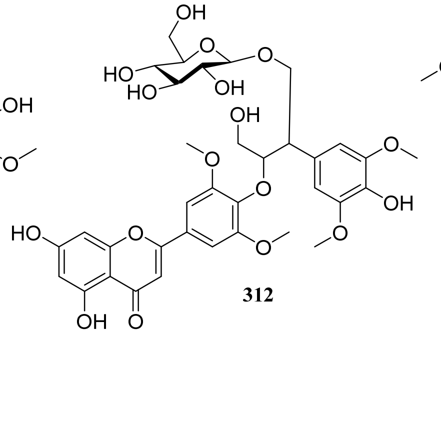

312

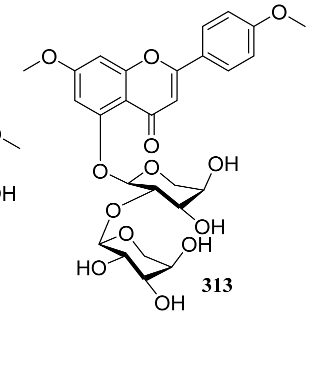

313

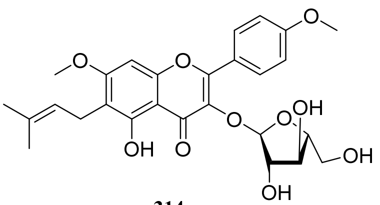

314

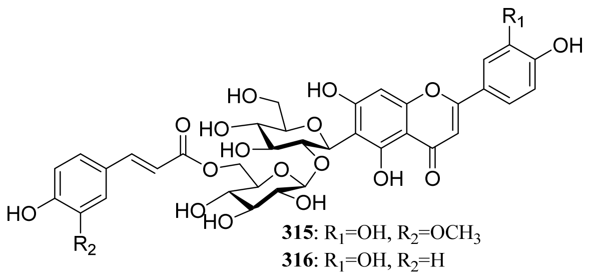

315: R_1=OH, R_2=OCH_3
316: R_1=OH, R_2=H
317: R_1=OCH_3, R_2=OCH_3

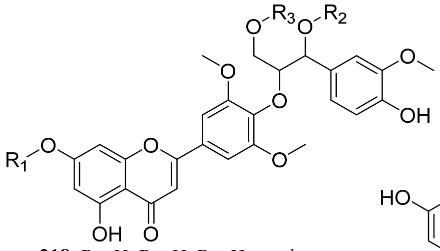

318: R_1=H, R_2=H, R_3=H, *erythro*
319: R_1=H, R_2=H, R_3=H, *threo*
320: R_1=Glc, R_2=H, R_3=H, *erythro*
321: R_1=Glc, R_2=H, R_3=H, *threo*
322: R_1=H, R_2=Glc, R_3=H, *erythro*
323: R_1=H, R_2=Glc, R_3=H, *threo*
324: R_1=H, R_2=H, R_3=Glc, *erythro*

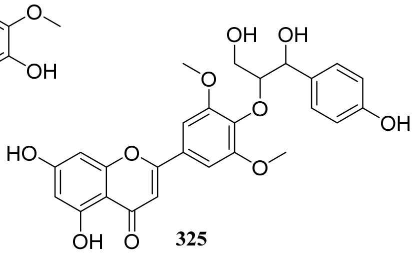

325

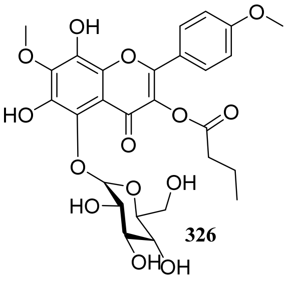

326

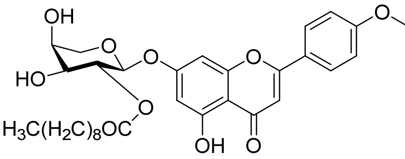

327

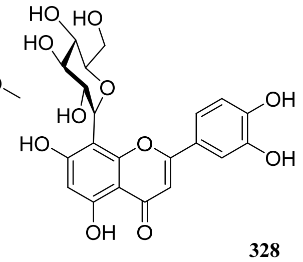

328

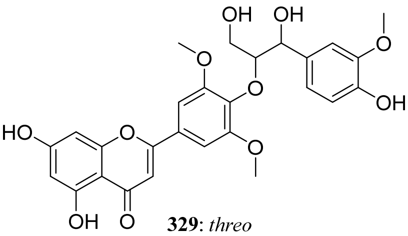

329: *threo*
330: *erythro*

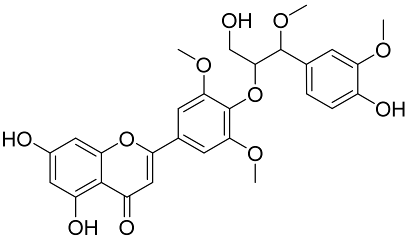

331: *threo*

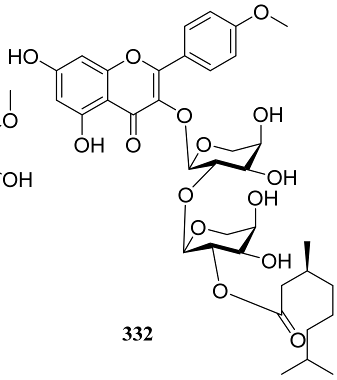

332

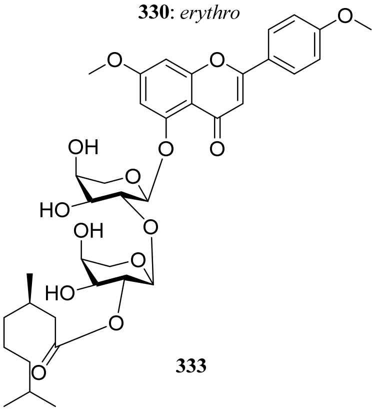

333

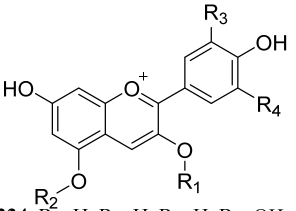

334: R_1=H, R_2=H, R_3=H, R_4=OH
335: R_1=Glc, R_2=H, R_3=H, R_4=OH
336: R_1=Glc-Glc, R_2=H, R_3=H, R_4=OH
337: R_1=Gal, R_2=H, R_3=H, R_4=OH
338: R_1=Glc-Rha, R_2=H, R_3=H, R_4=OH
339: R_1=Rut, R_2=H, R_3=H, R_4=OH
340: R_1=Glc-xylose, R_2=H, R_3=H, R_4=OH
341: R_1=Glc, R_2=Glc, R_3=H, R_4=OH
342: R_1=H, R_2=H, R_3=OH, R_4=OH
343: R_1=H, R_2=H, R_3=OCH_3, R_4=OCH_3
344: R_1=H, R_2=H, R_3=H, R_4=H
345: R_1=Glc, R_2=Glc, R_3=H, R_4=H
346: R_1=H, R_2=H, R_3=OCH_3, R_4=H
347: R_1=Glc, R_2=H, R_3=OCH_3, R_4=H

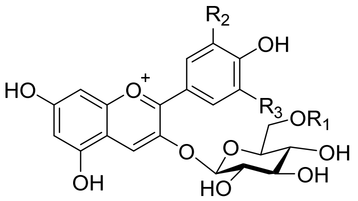

348: R_1=H, R_2=OCH_3, R_3=OH
349: R_1=H, R_2=OCH_3, R_3=OCH_3
350: R_1=Rha, R_2=OCH_3, R_3=H
351: R_1=Rha, R_2=H, R_3=H
352: R_1=H, R_2=OCH_3, R_3=OCH_3
353: R_1=comaroyl, R_2=OH, R_3=OH
354: R_1=comaroyl, R_2=OH, R_3=H

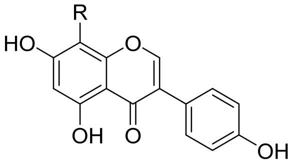

355: R=H
356: R=Glc

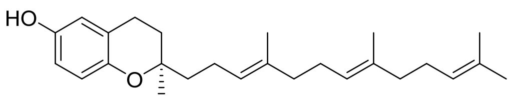

357

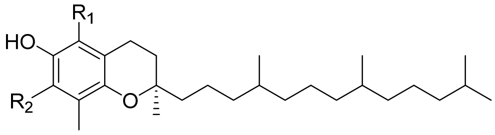

358: R_1=CH_3, R_2=CH_3
369: R_1=CH_3, R_2=H
360: R_1=H, R_2=CH_3
361: R_1=H, R_2=H

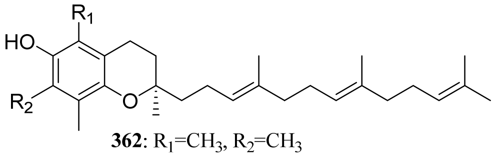

362: R_1=CH_3, R_2=CH_3
363: R_1=CH_3, R_2=H
364: R_1=H, R_2=CH_3
365: R_1=H, R_2=H

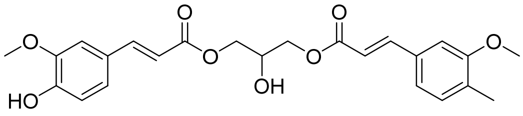

366

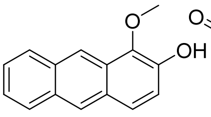

367

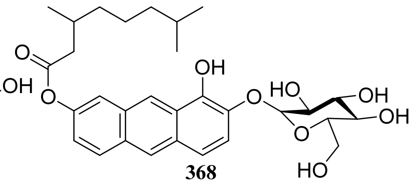

368

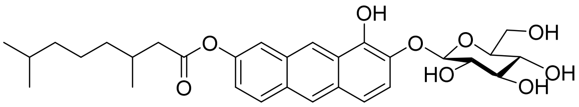

369

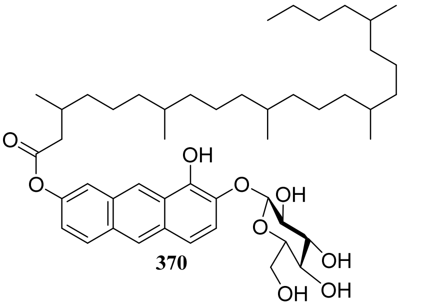

370

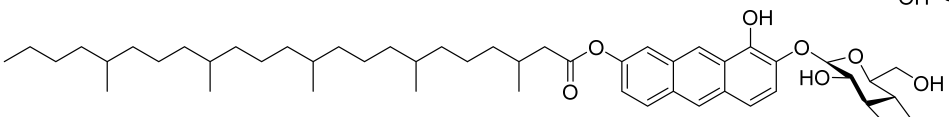

371

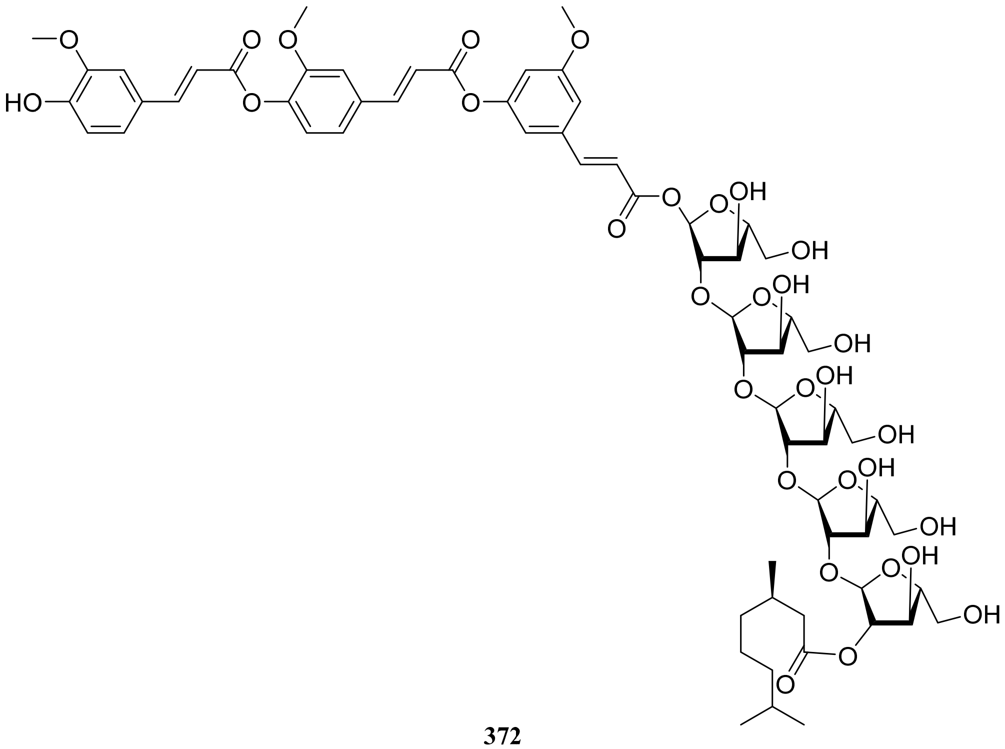

372

Figure S5. Chemical structures of rice phenolic compounds (**182–372**).

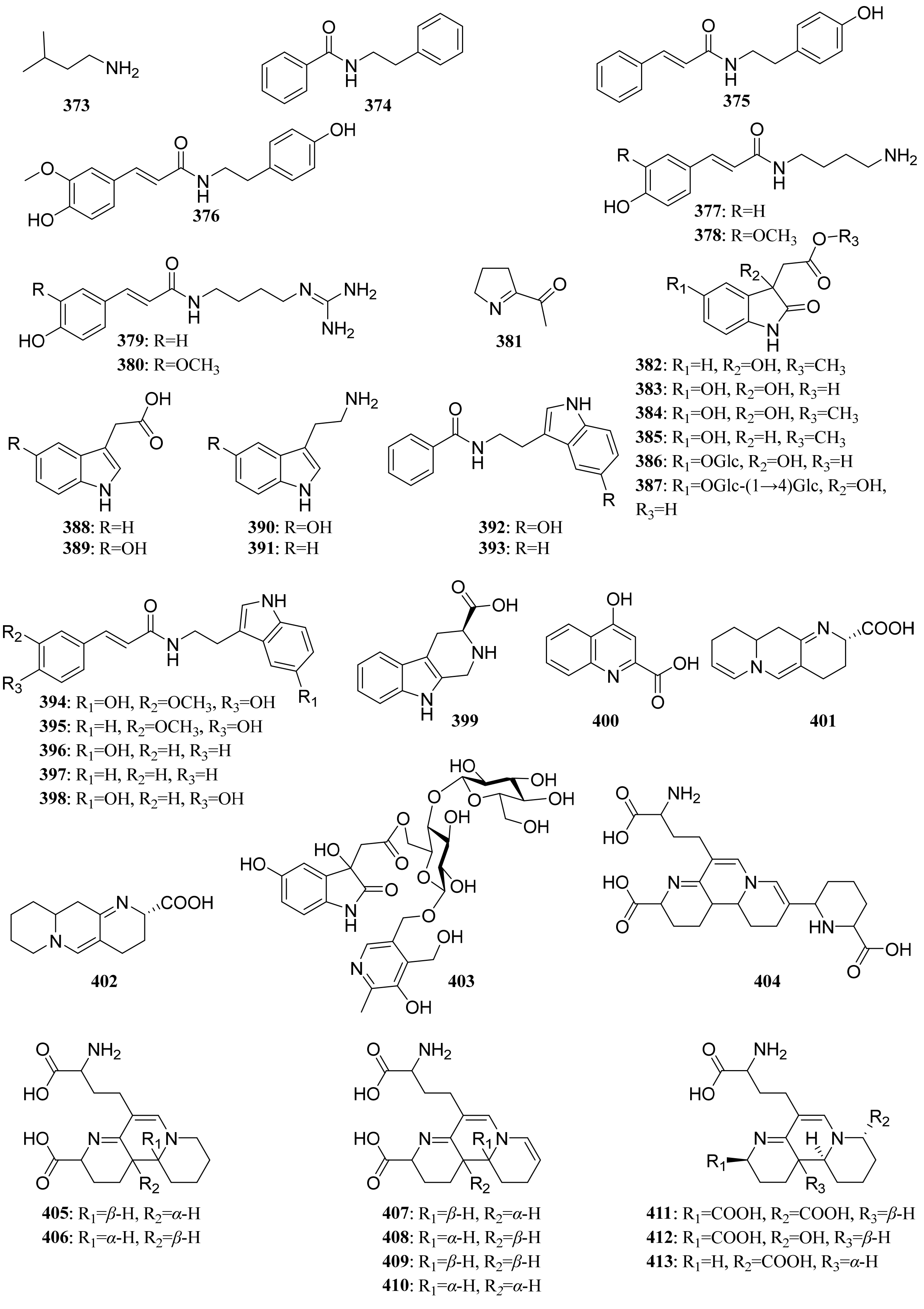

Figure S6. Chemical structures of rice alkaloids (**373–413**).

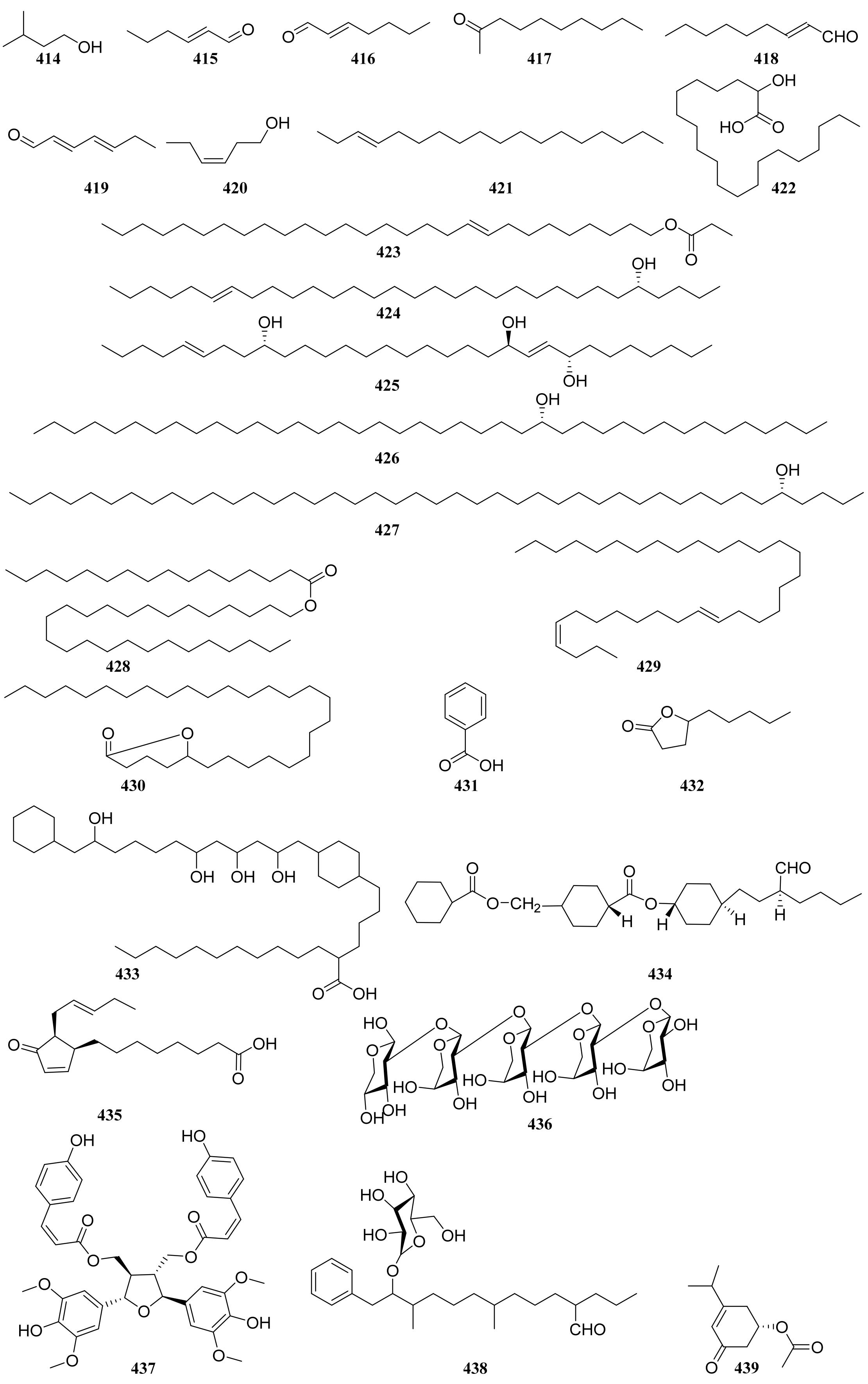

Figure S7. Chemical structures of other types of rice compounds (**414–439**).

Table S1. Terpenoids and steroids identified from rice (1–181).

No.	Name	Part of plant from which chemical was isolated	References
**1**	Myrcene	Seedlings	[28-29]
**2**	*trans*-β-Ocimene	Bran	[28]
**3**	Linalool	Leaves	[28-31]
**4**	Geraniol	Leaves	[32]
**5**	(*R*)-Limonene	Bran oil	[33]
**6**	(*S*)-Limonene	Leaves	[28]
**7**	Carveol	Bran	[28]
**8**	α-Terpinene	Seedlings	[29]
**9**	γ-Terpinene	Leaves	[34]
**10**	α-Phellandrene	Leaves	[35]
**11**	Terpinen-4-ol	Bran	[28]
**12**	Sabinene	Seedlings	[28-29]
**13**	α-Thujene	Seedlings	[29]
**14**	1,4-Cineol	Bran	[28]
**15**	α-Pinene	Seedlings	[29]
**16**	β-Pinene	Bran	[28]
**17**	Camphene	Bran	[28]
**18**	Camphor	Bran	[28]
**19**	*cis*-Linalool oxide	Bran	[28]
**20**	*trans*-Linalool oxide	Bran	[28]
**21**	Fenchyl acetate	Bran	[28]
**22**	(*E*)-β-Farnesene	Leaves	[36]
**23**	(*E*)-Nerolidol	Leaves	[36]
**24**	Orizaterpenyl benzoate	Hulls	[37]
**25**	α-Elemene	Bran	[28]
**26**	β-Elemene	Seedlings	[29]
**27**	(*Z*)-α-Bergamotene	Leaves	[30]
**28**	(*E*)-γ-Bisabolene	Leaves	[30]
**29**	α-Curcumene	Leaves	[30]
**30**	γ-Curcumene	Leaves	[30]
**31**	α-Zingiberene	Leaves	[30]
**32**	Germacrene D	Leaves	[30]
**33**	α-Humulene	Leaves	[30]
**34**	β-Caryophyllene	Leaves	[30]
**35**	(*E*)-β-Caryophyllene	Leaves	[30]

**36**	α-Chamigrene	Leaves	[30]
**37**	α-Cuprenene	Shoots	[38]
**38**	7-*epi*-α-Selinene	Bran	[28]
**39**	Valencene	Leaves	[30]
**40**	γ-Muurolene	Leaves	[30]
**41**	α-Cadinene	Leaves	[30]
**42**	α-Ylangene	Bran	[28]
**43**	α-Copaene	Leaves	[28-30]
**44**	β-Gurjunene	Leaves	[30]
**45**	Viridiflorene	Leaves	[30]
**46**	α-Gurjunene	Bran	[28]
**47**	Italicene	Leaves	[30]
**48**	Cyclosativene	Seedlings	[29]
**49**	β-Ionone	Stems	[39]
**50**	Orizanor-diterpenyl benzoate	Hulls	[37]
**51**	Orizaditerpenyl benzoate	Hulls	[37]
**52**	5-Deoxo-*ent*-10-oxodepressin	Leaves	[17]
**53**	5-Dihydro-*ent*-10-oxodepressin	Leaves	[17]
**54**	*ent*-10-Oxodepressin	Leaves	[40]
**55**	20-Epoxy-3α-hydroxy-8,11,13-abietatrie-7-one=3β,20β-Epoxy-3α-hydroxy-5α-abieta-8,11,13-trien-7-one	Hulls, Husks	[41-42]
**56**	Phytocassane A	Leaves, Stems	[18]
**57**	Phytocassane B	Leaves, Stems	[18]
**58**	Phytocassane C	Leaves, Stems	[18]
**59**	Phytocassane D	Leaves, Stems	[18]
**60**	Phytocassane E	Cultured rice cells	[19]
**61**	Phytocassane F	Leaves	[20]
**62**	Oryzalexin S	Leaves	[43-44]
**63**	Stemar-13-en-2α-ol	Leaves	[20]
**64**	Stemar-13-ene	Leaves	[45]
**65**	Oryzalexin A	Leaves, Roots	[21]
**66**	Oryzalexin B	Leaves	[23]
**67**	Oryzalexin C	Leaves	[22, 184]
**68**	Oryzalexin D	Leaves	[22, 184]
**69**	Oryzalexin E	Leaves	[185]
**70**	Oryzalexin F	Leaves	[49]
**71**	*ent*-Sandaraco-pimaradiene	Leaves	[48]
**72**	3α-Hydroxy-*ent*-sandaracopimaradiene	Leaves	[48]
**73**	9β*H*-pimara-7,15-diene	Leaves	[47]
**74**	9β-Pimara-7,15-diene-3β,6β,19-triol	Leaves	[20]
**75**	Momilactone E	Roots	[23]

**76**	Momilactone B	Root exudates, Coleoptiles, Seedlings, Bran	[8, 24-26, 50, 205]
**77**	Momilactone A	Coleoptiles, Bran	[8, 24-26]
**78**	Momilactone C	Bran	[12]
**79**	Momilactone D	Bran	[23]
**80**	4,6-Epoxy-3*β*-hydroxy-9*β*-pimara-7,15-diene=6β,19β-Epoxy-3β-hydroxy-5*α*,9β-pimara-7,15-diene	Hulls, Husks	[41-42]
**81**	2-((*E*)-3-(4-Hydroxy-3-methoxyphenyl) allylidene) momilactone A	Coleoptiles, Bran, Root exudates	[8, 24-26, 42]
**82**	Sandaracopimaradien-3-one	Roots	[23]
**83**	Oryzalic acid A	Leaves	[180]
**84**	Oryzalic acid B= *ent*-15-Hydroxy-2,3-secokauren	Leaves	[178]
**85**	*ent*-2,3,15-Trihydroxy- kaurane	Leaves	[178]
**86**	Oryzalide A= *ent*-15,16-Epoxy-1*α*-hydroxy-2-oxa-kauran-3-one	Leaves, Roots	[179-180]
**87**	Oryzalide B	Leaves	[180]
**88**	*ent*-15,16-Epoxy-3-oxa-kauran-2-one	Leaves	[21]
**89**	Gibberellin A_1	Whole plant	[46]
**90**	Gibberellin A_4	Whole plant	[46]
**91**	Gibberellin A_{19}	Whole plant	[46]
**92**	*ent-7-oxo*-Kaur-15-en-18-oic acid	Hulls	[42]
**93**	Orizaterpenol	Hulls	[37]
**94**	*ent*-3*α*,16*α*-Dihydroxy-kauran-19-oic acid	Husks	[10]
**95**	*ent*-15,16-Epoxy-2,3-dihydroxy- kaurane	Leaves	[178]
**96**	*ent*-15,16-Epoxy-kauran-3-one	Leaves	[21]
**97**	*ent*-15,16-Epoxy-kauran-2,3-dione=Oryzadione	Leaves	[21]
**98**	*ent*-15,16-Epoxy-3β-hydroxy-kauran-2-one	Leaves	[21]
**99**	*ent*-15,16-Epoxy-3β-myristoyloxy-kauran-2-one	Leaves	[21]
**100**	*ent*-15,16-Epoxy-3β-palmitoyloxy-kauran-2-one	Leaves	[21]
**101**	*ent*-15,16-Epoxy-3*α*-palmitoyloxy-kauran-2-one	Leaves	[21]
**102**	4-(Hydroxymethyl)-7-(2,2,6-trimethylcyclohexyl)-heptyl 2,6,6-trimethyl-1,3-cyclohexadiene-1- carboxylate	Hulls	[27]
**103**	Orizaterpenoid	Hulls	[42]
**104**	Citrostadienol	Bran	[53-54]
**105**	*trans*-Ferulate-citrostadienol	Bran	[54]
**106**	*cis*-Ferulate-citrostadienol	Bran	[54]
**107**	*trans*-Caffeate-citrostadienol	Bran	[54]
**108**	Gramisterol	Bran	[55]
**109**	*trans*-Ferulate-gramisterol	Bran	[54]
**110**	*cis*-Ferulate-gramisterol	Bran	[54]
**111**	Cycloeucalenol	Bran	[55]

**112**	*trans*-Ferulate-cycloeucalenol	Bran	[56]
**113**	*cis*-Ferulate-cycloeucalenol	Bran	[54]
**114**	Cycloartanol	Bran	[53, 67]
**115**	*trans*-Ferulate-cycloartanyl	Bran oil	[57]
**116**	Lanast-7,9(11)-dien-3*α*,15*α*-diol-3*α*-D-glucofuranoside	Husk	[58]
**117**	Cyclobranol	Bran	[59]
**118**	3β-24-Methylenecycloartan-3-ol	Bran	[68]
**119**	*trans*-Ferulate-24-methylenecycloartanyl	Bran oil	[60]
**120**	*cis*-Ferulate-24-methylenecycloartanyl	Bran oil	[60]
**121**	Cycloart-23Z-ene-3β,25-diol-3β-*trans*-ferulate=Cycloart-23-ene-3,25-diol,3-*O*-(4-hydroxy-3-methoxy-*E*-cinnamoyl)	Bran, Bran oil	[61-62]
**122**	(24*S*)-Cycloart-25-ene-3β,24-diol-3β-*trans*-ferulate	Bran	[61]
**123**	(24*R*)-Cycloart-25-ene-3β,24-diol-3β-*trans*-ferulate	Bran	[61]
**124**	Cycloartenol	Bran	[53, 67]
**125**	*trans*-Caffeate-cycloartenol	Seeds	[63]
**126**	*trans*-Ferulate-cycloartenol	Bran	[56, 61]
**127**	*cis*-Ferulate-cycloartenol	Bran	[56]
**128**	*trans*-Ferulate-cycloartenyl	Bran oil	[68]
**129**	5-Hydroxy-7,4'-dimethoxyflavone-5-*O*-*β*-D-arabinofuranosyl-(2''→1''')-*O*-*β*-D-arabinopyranosyl-2'''-*O*-lanost-5-ene	Straw, Leaves	[64]
**130**	Orizalanosterolide A	Hulls	[65]
**131**	Orizalanosterolide B	Hulls	[65]
**132**	Lupeol	Bran	[55]
**133**	Lupenone	Bran	[55]
**134**	Ursolic acid 28-*O*-*β*-D-xylofuranosyl-(2'→1'')-*O*-*β*-D-xylofuranosyl-(2''→1''')-*O*-*β*-D-xylopyranoside	Straw, Leaves	[64]
**135**	Bayogenin 3-*O*-β-D-cellobioside	Leaves	[66]
**136**	Bayogenin	Leaves	[66]
**137**	Fucosterol	Bran	[55]
**138**	Sitostanol	Germinating seeds	[63]
**139**	Campestanol	Germinating seeds	[63]
**140**	*trans*-Ferulate-campestanol	Bran	[56]
**141**	*trans*-Ferulate-stigmastanol	Bran	[56]
**142**	*cis*-Ferulate-stigmastanol	Bran	[54]
**143**	Stigmasterol	Bran, Seedlings	[53, 212]
**144**	7-Ketostigmasterol	Husk	[69]
**145**	*trans*-Ferulate-stigmasterol	Bran	[56]
**146**	*cis*-Ferulate-stigmasterol	Bran	[54]
**147**	Cholesterol	Germinating seeds	[63]
**148**	*cis*-Ferulate-24-methyl cholesterol	Bran	[53]
**149**	*cis*-Ferulate-24-methylene cholesterol	Bran	[54]

**150**	*trans*-Ferulate-24-methylene cholesterol	Bran	[56]
**151**	Campesterol	Bran, Seedlings	[55]
**152**	24-Methylene ergosta-5-en-3β-ol=24-Methylenecholesterol	Bran	[54]
**153**	Δ^5-Avenasterol	Germinating seeds	[63]
**154**	Sitosterol=β-Sitosterol	Bran, Seedlings	[53]
**155**	7α-Hydroxy sitosterol	Bran	[70]
**156**	7β-Hydroxy sitosterol	Bran	[70]
**157**	Schleicheol 2	Bran	[70]
**158**	*trans*-Caffeate-campesterol	Bran	[56]
**159**	*trans*-Ferulate-campesterol	Bran	[56]
**160**	*trans*-Ferulate-cholesteryl	Bran oil	[68]
**161**	*trans*-Ferulate-24-methylene cycolartanol	Bran	[56]
**162**	*trans*-Ferulate-sitosterol =Feruloyl-β-sitosterol	Bran, Bran oil	[56, 74]
**163**	*cis*-Ferulate-sitosterol	Bran	[56]
**164**	β-sitosterol-β-D-glucoside	Hulls	[6]
**165**	D-Glucopyranosyl-(β1→4)-D-glucopyranosyl-(β1→3')-β-sitosterol	Bran, Hulls	[71]
**166**	D-Glucopyranosyl-(β1→3)-D-glucopyranosyl-(β1→3')-β-sitosterol	Bran, Hulls	[71]
**167**	D-Glucopyranosyl-(β1→4)-D-glucopyranosyl-(β1→4)-D-glucopyra-nosyl-(β1→3')-β-sitosterol	Bran, Hulls	[71]
**168**	Cellotetraosylsitosterol	Bran	[72]
**169**	Cellopentaosylsitosterol	Bran	[72]
**170**	24-Methylene ergosta-7-en-3β-ol	Bran	[55]
**171**	Δ^7-Avenasterol	Germinating seeds	[63]
**172**	Δ^7-Campestenol	Germinating seeds	[63]
**173**	Δ^7-Stigmastenol	Germinating seeds	[63]
**174**	Δ^7-*trans*-Ferulate-campesterol	Bran	[61]
**175**	Δ^7-*trans*-Ferulate-sitosterol	Bran	[56]
**176**	Stigmast-5-en-3α-26-diacetate	Hulls	[6]
**177**	14-Methylstigmast-9(11)-en-3-ol,3-*O*-β-D-glucopyranoside	Hulls	[27]
**178**	3-Benzyloxy-stigmast-7-one-22-en-19-oic acid 29-ethyleneglycol ether	Seed husks	[69]
**179**	Stigmastanol-3β-*p*-glyceroxy dihydrocoumaroate=Stigmastanol-3β-*p*-butanoxydihydrocoumaroate	Hulls, Husk	[58]
**180**	Stigmastanol-3β-*p*-butanoxy dihydrocoumaroate=Stigmastanol-3β-*p*-glyceroxydihydrocoumaroate	Hulls, Husk	[58]
**181**	3,6,7,22-Tetrahydroxycholest-11-en-24-one,3-*O*-(9Z-hexadecenoyl)	Hulls	[73]

Table S2. Phenolic compounds identified from rice (182–372).

No.	Name	Part of plant from which chemical was isolated	References
**182**	Ethyl benzoate	Bran	[33]
**183**	*p*-Hydroxybenzaldehyde	Husk, Bran	[77-78]
**184**	*p*-Hydroxybenzoic acid=4-Hydroxybenzoic acid	Husk, Bran, Root exudate	[77, 79]
**185**	*p*-Hydroxy phenyl acetaldehyde	Husk, Bran	[77]
**186**	*p*-Hydroxy phenyl acetic acid=4-Hydroxyphenylacetic acid	Husk, Bran	[77]
**187**	2-Hydroxyphenylacetic acid	Bran	[79]
**188**	Vanillic aldehyde	Bran	[78]
**189**	Vanillic acid	Husk, Bran, Root exudate	[77, 83, 79]
**190**	Caffeic acid	Endosperm, Bran, Embryo, Root exudate, Husk	[80]
**191**	Methyl caffeate	Bran	[78]
**192**	Eugenol	Husks, Straw	[81]
**193**	*cis*-Isoeugenol	Husks, Straw	[81]
**194**	*trans*-Ferulic acid	Husks, Straw	[80, 84]
**195**	*trans*-Ferulic acid methyl ester	Bran	[78]
**196**	*trans*-Coniferaldehyde	Bran	[170]
**197**	*cis*-Ferulic acid	Husks, Straw	[78]
**198**	*cis*-Ferulic acid methyl ester	Bran	[78]
**199**	Guaicol	Husks, Straw	[81]
**200**	*trans*-Isoeugenol	Husks, Straw	[81]
**201**	4-Propylguaiacol	Husks, Straw	[81]
**202**	2-Propiovanillone	Husks, Straw	[81]
**203**	Sinapic acid	Grains	[78]
**204**	Syringol	Husks, Straw	[81]
**205**	4-Methylsyringol	Husks, Straw	[81]
**206**	4-Ethylsyringol	Husks, Straw	[81]
**207**	4-Allylsyringol	Husks, Straw	[81]
**208**	4-Vinylsyringol	Husks, Straw	[81]
**209**	*trans*-4-Propenylsyringol	Husks, Straw	[81]
**210**	Syringaldehyde	Grains	[72]
**211**	Syringic acid	Endosperm, Bran, Embryo, Root exudate, Husk, Grains	[73]
**212**	Acetosyringone	Husks, Straw	[81]
**213**	Salicylic acid 2-*O*-β-D-glucopyranoside	Leaves	[82]
**214**	Protocatechuic acid	Endosperm, Bran, Embryo	[73]

**215**	Gallic acid	Endosperm, Bran, Embryo, Root exudate, Husk, Grains	[77, 80]
**216**	Cinnamic acid	Husk, Bran	[77]
**217**	*o*-Coumaric acid	Endosperm, Bran, Embryo, Root exudate	[73]
**218**	*m*-Coumaric acid	Grains	[78]
**219**	*p*-Coumaric acid	Grains	[83-84]
**220**	Chalcone	Grain hulls	[92]
**221**	Chlorogenic acid	Endosperm, Bran, Embryo	[80]
**222**	Caffeoylquinic acid methyl ester	Grains	[83]
**223**	3-*O*-*p*-Coumaroyl quinic acid	Grains	[83]
**224**	3-*O*-Feruloylquinic acid	Leaves	[82]
**225**	Oryzafuran	Bran	[85]
**226**	Salicylic acid β-D-glucoside	Grains	[86]
**227**	*p*-Hydroxy methyl benzoate glucoside	Bran	[78]
**228**	2-Hydroxy 5-[(3*S*)-3-hydroxybutyl] phenyl β-D-glucoside (HHPG)	Bran	[87]
**229**	1-*O*-Feruloyl-β-D-glucose	Leaves	[82]
**230**	1-*O*-Sinapoyl-β-D-glucose	Leaves	[82]
**231**	Phloretin 2'-*O*-β-D-glucopyranoside	Grain	[94]
**232**	Phloretin	Grain	[94]
**233**	Hesperetin	Grain hulls	[92]
**234**	Epicatechin	Rice flour	[93]
**235**	Catechin	Rice flour	[93]
**236**	Dihydromyricetin	Grain hulls	[92]
**237**	Eriodictyol	Grain hulls	[92]
**238**	Dihydroquercetin ((2R,3R)-2-(3,4-dihydroxyphenyl)-3,5,7-trihydroxy-2,3-dihydrochromen-4-one)	Grain hulls	[92]
**239**	Dihydrokaempferol	Grain hulls	[92]
**240**	Luteolin	Grain	[94]
**241**	Hispidulin	Grain	[94]
**242**	Apigenin	Leaves	[92]
**243**	2'-(1''β-hydroxypropyl)-7,4'-dimethoxyapigenin	Seed husks	[69]
**244**	Naringenin	Leaves	[95]
**245**	4'-Methoxyapigenin	Leaves, straw	[96]
**246**	Sakuranetin	Leaves	[97]
**247**	Sternbin	Leaves	[98]
**248**	Sakuranetin-4'-*O*-β-D-xylopyranoside	Sheath	[99]
**249**	Naringenin-7-*O*-β-D-xylopyranoside	Sheath	[99]
**250**	Diosmetin	Grain	[94]
**251**	Narirutin	Grain	[94]

**252**	Hesperidin	Hesperidin	[93]
**253**	Butin-*O*-glucoside	Grain	[94]
**254**	5,7-Dihydroxy-4-methoxyflavone-7-*O*-β-D-arabinopyranoside	Leaves, Straw	[96]
**255**	Isorhamnetin-3-*O*-rhamnoside	Grain	[94]
**256**	Isorhamnetin-3-*O*-rutinoside	Grain	[94]
**257**	3'-*O*-Methyltaxifolin	Grain	[109]
**258**	3'-*O*-Methyltaxifolin-7-*O*-β-D-glucopyranoside	Grain	[109]
**259**	3'-*O*-Methyltaxifolin-4'-*O*-β-D-glucopyranoside	Grain	[109]
**260**	3'-*O*-Methyltaxifolin-5-*O*-β-D-glucopyranoside	Grain	[109]
**261**	Tricetin	Grain	[94]
**262**	Apigenin-7-*O*-rutinoside (Isorhoifolin)	Grain	[94]
**263**	Chrysoeriol 7-*O*-rutinoside	Grain	[83]
**264**	Luteolin-3',7-di-*O*-glucoside	Grain	[94]
**265**	Luteolin-7-*O*-glucoside (Cynaroside)	Grain	[94]
**266**	Apigenin-7-*O*-β-D-glucoside	Grain	[94]
**267**	Luteolin 7-*O*-rutinoside	Grain	[94]
**268**	Luteolin-6-*C*-glucoside (Isoorientin)	Grain	[94]
**269**	Luteolin-6-*C*-glucoside (Isovitexin)	Grain	[94]
**270**	Isoscoparin	Grain	[94]
**271**	Apigenin 6-*C*-α-L-arabinosyl-8-*C*-β-L-arabinoside	Leaves	[110]
**272**	Chrysoeriol 6-*C*-α-L-arabinosyl-8-*C*-β-L-arabinoside	Leaves	[110]
**273**	Vitexin 2''-*O*-glucoside (Apigenin 8-*C*-glucoside-2''-*O*-glucoside)	Leaves	[101]
**274**	Luteolin 6-*C*-(2''-*O*-β-D-glucopyranosyl)-α-L-arabinoside	Leaves	[110]
**275**	Schaftoside	Leaves	[108]
**276**	Isoschaftoside	Leaves	[108]
**277**	Apigenin-8-*C*-glucoside	Grain hulls	[92]
**278**	Kaempferol	Husk, Bran	[77, 100]
**279**	Kaempferol 3-*O*-robinobioside	Leaves	[92]
**280**	Kaempferol 3-*O*-rutinoside	Leaves	[92]
**281**	Myricetin	Rice flour	[93]
**282**	Quercetin	Rice flour	[93, 100]
**283**	Quercetin 3-*O*-glucoside	Rice flour	[93]
**284**	Quercetin 3-*O*-galactoside=Hyperoside	Rice flour	[93]
**285**	Qucertin 3-*O*-rutinoside=Rutin	Rice flour	[93]
**286**	Syringetin 3-*O*-β-D-glucopyranoside	Rice flour	[110]
**287**	Syringetin 3-*O*-rutinoside	Leaves	[110]
**288**	Kaempferol 3-*O*-β-d-(6''-*O*-(*E*)-*p*-coumaroyl)glucopyranoside	Grain hulls	[92]
**289**	Isoorientin 7,3'-dimethyl ether	Leaves	[110]

**290**	Swertisin	Leaves	[110]
**291**	5,6-Dihydroxy-7,4'-dimethoxyflavone-5-*O*-a-D-glucopyranoside	Leaves, Straw	[96]
**292**	Brassicin	Grain	[109]
**293**	Brassicin-4'-*O*-β-D-glucopyranoside	Grain	[109]
**294**	Isorhamnetin-4'-*O*-β-D-glucopyranoside	Grain	[109]
**295**	Isorhamnetin-7-*O*-β-D-cellobioside	Grain	[109]
**296**	Quercetin-7-*O*-Glucoside	Grain	[94]
**297**	Tricin=5,7,4'-Trihydroxy-3',5'-dimethoxyflavone	Bran	[111]
**298**	Tricin 7-*O*-β-D-glucopyranoside=5,4'-Dihydroxy-3',5'-dimethoxy-7-*O*-β-glucopyranosylflavone=Tricin 7-*O*-glucoside	Leaves	[110]
**299**	Tricin 5-*O*-β-D-glucopyranoside=7,4'-Dihydroxy-3',5'-dimethoxy-5-*O*-β-glucopyranosylflavone=Tricin 5-*O*-glucoside	Leaves	[110]
**300**	Tricin 7-*O*-rutinoside	Leaves	[110]
**301**	Tricin 7-*O*-neohesperidoside	Leaves	[110]
**302**	Tricin 7-*O*-(2''-*O*-β-D-glucopyranosyl)-β-D-glucuronopyranoside	Leaves	[110]
**303**	Tricin 4'-*O*-glucoside	Grain	[94]
**304**	Isoscoparin 2-*O*-(6-(*E*)-feruloyl)-glucopyranoside	Leaves	[110]
**305**	Isoscoparin 2''-*O*-(6'''-(*E*)-*p*-coumaroyl)-glucopyranoside	Leaves	[110]
**306**	Isoscoparin 2''-*O*-glucoside (Chrysoeriol 6-*C*-glucoside-2''-*O*-glucoside)	Leaves	[101]
**307**	Isovitexin 2''-*O*-(6'''-(*E*)-feruloyl)-glucopyranoside	Leaves	[110]
**308**	Isovitexin 2''-*O*-(6'''-(*E*)-*p*-coumaroyl)-glucopyranoside	Leaves	[110]
**309**	Isoscoparin-2''-*O*-β-d-glucopyranoside	Leaves	[110]
**310**	Tricin 7-*O*-(6''-*O*-malonyl)-β-D-glucopyranoside	Leaves	[110]
**311**	Tricin 7-*O*-(6''-(*E*)-sinapoyl)-β-D-glucopyranoside	Leaves	[110]
**312**	Tricin 4'-*O*-(threo-β-syringylglyceryl) ether 7''-*O*-β-D-glucopyranoside	Leaves	[110]
**313**	7,4'-Dimethoxy-5-hydroxyflavone-5-*O*-*α*-D-arabinopyranosyl-(2''→1''')-*O*-*α*-D-arabinopyranoside	Leaves, Straw	[96]
**314**	5-Hydroxy-6-isoprenyl-7,4'-dimethoxyflavonol-3-*O*-β-D-arabinofuranoside	Leaves, Straw	[96]
**315**	Isoorientin 2''-*O*-(6'''-(*E*)-feruloyl)glucoside	Leaves	[146]
**316**	Isoorientin 2''-*O*-(6'''-(*E*)-*p*-coumaroyl)glucoside	Leaves	[146]
**317**	Isoscoparin 2''-*O*-(6'''-(*E*)-feruloyl)glucoside	Leaves	[146]
**318**	Tricin 4'-O-(*erythro*-β-guaiacylglyceryl) ether	Bran	[112]
**319**	Tricin 4'-*O*-(*threo*-β-guaiacylglyceryl) ether	Bran	[110]
**320**	Tricin 4'-*O*-(*erythro*-β-guaiacylglyceryl) ether 7-*O*-β-D-glucopyranoside	Leaves	[110]

**321**	Tricin 4'-*O*-(*threo*-β-guaiacylglyceryl) ether 7-*O*-β-D-glucopyranoside	Leaves	[110]
**322**	Tricin 4'-*O*-(*erythro*-β-guaiacylglyceryl) ether 7''-*O*-β-D-glucopyranoside	Leaves	[110]
**323**	Tricin 4'-*O*-(*threo*-β-guaiacylglyceryl) ether 7''-*O*-β-D-glucopyranoside	Leaves	[110]
**324**	Tricin 4'-*O*-(*erythro*-β-guaiacylglyceryl) ether 9''-*O*-β-D-glucopyranoside	Leaves	[110]
**325**	Tricin 4'-*O*-(*threo*-β-4-hydroxyphenylglyceryl) ether	Leaves	[110]
**326**	3-Butanoyl-5,6,8-trihydroxy-7,4'-dimethoxyflavonol-5-*O*-B-Dglucopyranoside	Leaves, Straw	[96]
**327**	5,7-Dihydroxy-4'-methoxyflavone-7-*O*-β-D-arabinopyranosyl-2''-n-decan-1'''-oate	Leaves, Straw	[96]
**328**	Orientin	Grain	[94]
**329**	Salcolin A	Grain	[102]
**330**	Salcolin B	Grain	[102]
**331**	Salcolin C (tricin-4'-*O*-[erythro-β-guaiacyl-(7''-O-methyl)-glyceryl] ether)	Grain	[102]
**332**	5,7-Dihydroxy-4'-methoxyflavonol-3-*O*-β-D-arabinopyranosyl-(2''→1''')-*O*-β-D-arabinopyrnosyl-2'''-*O*-3'''', 7''''-dimethylnonan-1''''-oate	Straw, Leaves	[64]
**333**	5-Hydroxy-7,4'-dimethoxyflavone-5-*O*-*α*-D-arabinopyranosyl-(2''→1''')-*O*-*α*-D-arabinopyranosyl-2'''-3'''',7''''-dimethylnonan-1''''-oate	Straw, Leaves	[64]
**334**	Cyanidin	Bran	[67, 78]
**335**	Cyanidin 3-*O*-glucoside	Bran	[87, 107]
**336**	Cyanidin 3-*O*-gentiobioside	Bran	[87, 107]
**337**	Cyanidin-3-galactoside	Bran	[117]
**338**	Cyanidin-3-*O*-rhamnoglucoside	Bran	[103]
**339**	Cyanidin 3-*O*-rutinoside	Kernels	[104]
**340**	Cyanidin 3-*O*-sambubioside	Black rice kernels	[106]
**341**	Cyanidin 3,5-diglucoside	Bran	[104]
**342**	Delphinidin	Bran	[67]
**343**	Malvidin	Bran	[67]
**344**	Pelargonidin	Bran	[67]
**345**	Pelargonidin 3,5-*O*-diglucoside	Pigmented rice	[90]
**346**	Peonidin	Black rice kernels	[20]
**347**	Peonidin 3-*O*-glucoside	Bran	[78, 87, 107]

**348**	Petunidin 3-*O*-glucoside	Bran	[105]
**349**	Malvidin 3-*O*-glucoside	Bran	[105]
**350**	Peonidin-3-*O*-rutinoside	Bran	[84]
**351**	Pelargonidin-3-*O*-rutinoside	Grain	[94]
**352**	Malvidin 3-*O*-glucoside	Grain hulls	[92]
**353**	Delphinidin-3-*O*-(6''-*p*-Coumaroylglucoside)	Grain	[94]
**354**	Cyanidin-3-*O*-(6''-*p*-Coumaroylglucoside)	Grain	[94]
**355**	Genistein	Grain hulls	[92]
**356**	Genistein 8-C-glucoside	Grain hulls	[92]
**357**	3,4-Dihydro-2-methyl-2-(4,8,12-trimethyl-3,7,11-tridecatrienyl)-2H-1-benzopyran-6-ol (R&S form)	Bran	[113]
**358**	α-Tocopherol	Bran	[114]
**359**	β-Tocopherol	Bran	[114]
**360**	γ-Tocopherol	Bran	[114]
**361**	δ-Tocopherol	Bran	[114]
**362**	α-Tocotrienol	Bran	[114]
**363**	β-Tocotrienol	Bran	[114]
**364**	γ-Tocotrienol	Bran	[114]
**365**	δ-Tocotrienol	Bran	[114]
**366**	1,3-*O*-Diferuloylglycerol	Leaves	[82]
**367**	Orizaanthracenol=1-Methoxyanthracen-2-ol	Hulls	[115]
**368**	Anthracenetriol, 7-*O*-(3,7-dimethyloctanoyl), 2-*O*-β-D-glucopyranosid	Hulls	[116]
**369**	1-Hydroxy-7-((2S,3R,4R,5S)-2'',3'',4''-trihydroxy-5''-(hydroxymethyl)tetrahydro-2*H*-pyran-1-yloxy)anthracen- 2-yl 3',7'-dimethyloctanoate	Hulls	[115]
**370**	Anthracenetriol, 7-*O*-(3,7,11,15,19- pentamethyltricosanoyl), 2-*O*-β-D-glucopyranosid	Hulls	[116]
**371**	1-Hydroxy-7-((2*S*,3*R*,4*R*,5*S*)-2'',3'',4''-trihydroxy-5''-(hydroxymethyl)tetrahydro-2*H*-pyran-1-yloxy)anthracen- 2-yl 3',7',11',15',19'-pentamethyltricosanoate	Hulls	[115]
**372**	4',4''-Diferuloxy feruloyl-*O*-*α*-D-arabinopyranosyl-(2a→1b)-*O*-*α*-D-arabinopyranosyl-(2b→1c)-*O*-*α*-D-arabinopyranosyl-(2c→1d)-*O*-*α*-D-arabinopyranosyl-(2d→1e)-*O*-*α*-D-arabinopyranosyl-2e-3''', 7'''-dimethylnonan-1'''-oate	Straw, Leaves	[64]

Table S3. Alkaloids identified from rice (373–413).

No.	Name	Part of plant from which chemical was isolated	References
**373**	Isopentylamine	Whole rice plant	[119]
**374**	*N*-Benzoyltyramine	Leaves	[120-121]
**375**	*N*-*trans*-Cinnamoyltyramine	Leaves	[120]
**376**	*N*-Feruloyltyramine (FerTyr)	Bran	[122]
**377**	*N*-*p*-Coumaroylputrescine	Leaves	[123]
**378**	*N*-Feruloylputrescine	Leaves	[120]
**379**	*N*-*p*-Coumaroylagmatine	Leaves	[120]
**380**	*N*-Feruloylagmatine	Leaves	[120]
**381**	2-Acetyl-1-pyrroline	Grains	[124]
**382**	Coixlactam B	Bran	[125-126]
**383**	2,3-Dihydro-3,5-dihydroxy-2-oxo-1*H*-indole-3-acetic acid	Bran	[125]
**384**	2,3-Dihydro-3,5-dihydroxy-2-oxo-1H-indole-3-acetic acid, methyl ester	Bran	[125]
**385**	2,3-Dihydro-5-hydroxy-2-oxo-1H-indole-3-acetic acid, methyl ester	Bran	[125]
**386**	2,3-Dihydro-3-dihydroxy-2-oxo-1H-indole-3-acetic acid, 5-*O*-β-D-glucopyranoside (R form)	Bran	[125]
**387**	2,3-Dihydro-3-dihydroxy-2-*oxo*1H-indole-3-acetic acid, 5-*O*-[β-D-glucopyranosyl-(1→4)-β-D-glucopyranoside]	Bran	[125]
**388**	Indole 3-acetic acid	Whole rice plant	[127]
**389**	5-Hydroxyindole-3-acetic acid	Leaves	[128]
**390**	Serotonin=5-Hydroxytryptamine	Leaves	[128]
**391**	Tryptamine	Leaves	[128]
**392**	*N*-Benzoylserotonin	Leaves	[120]
**393**	*N*-Benzoyltryptamine	Leaves	[121]
**394**	N-Feruloylserotonin	Leaves	[120]
**395**	*N*-Feruloyltryptamine	Leaves	[128]
**396**	*N*-*trans*-Cinnamoylserotonin	Leaves	[120]
**397**	*N*-*trans*-Cinnamoyltryptamine	Leaves	[120]
**398**	*N*-*p*-Coumaroylserotonin	Leaves	[121, 204]
**399**	Lycoperodine-1	Leaves	[82]
**400**	Kynurenic acid	Leaves	[82]
**401**	Oryzadiamine A	Yellow grain	[129]
**402**	Oryzadiamine B	Yellow grain	[129]
**403**	5-[6-(5-Hydroxydioxindole-3- acetyl)-β-cellobiosyl]pyridoxine	Bran	[180]
**404**	Oryzamutaic acid A	Endosperm	[131]

**405**	Oryzamutaic acid B	Endosperm	[132]
**406**	Oryzamutaic acid C	Endosperm	[132]
**407**	Oryzamutaic acid D	Endosperm	[132]
**408**	Oryzamutaic acid E	Endosperm	[132]
**409**	Oryzamutaic acid F	Endosperm	[132]
**410**	Oryzamutaic acid G	Endosperm	[132]
**411**	Oryzamutaic acid H	Endosperm	[133]
**412**	Oryzamutaic acid I	Endosperm	[133]
**413**	Oryzamutaic acid J	Endosperm	[133]

Table S4. Other types of compounds identified from rice (414–439).

No.	Name	Part of plant from which chemical was isolated	References
**414**	3-Methyl-1-butanol	Kernels	[33]
**415**	(*E*)-2-Hexenal	Kernels	[33]
**416**	2-Heptenal	Kernels	[33]
**417**	2-Decanone	Kernels	[33]
**418**	*cis*-2-Nonenal	Kernels	[33]
**419**	(*E*,*E*)-2,4-Heptadienal	Whole plants	[135]
**420**	(*Z*)-3-Hexen-1-ol	Leaves	[30]
**421**	*n*-Hexatriacont-15-ene	Straw, Leaves	[64]
**422**	2-Hydroxyeicosanoic acid (R form)	Bran	[136]
**423**	*n*-Octacos-9-enyl propionate	Hulls	[137]
**424**	*n*-Hentriacont-25-en-5*α*-ol	Hulls	[137]
**425**	*n*-Hexetriacont- 9,26-dien-8*α*,11β,23*α*-triol	Hulls	[137]
**426**	*n*-Tetracontan-15*α*-ol	Hulls	[137]
**427**	*n*-Tritetracontan-5*α*-ol	Hulls	[137]
**428**	Hexacosyl ester	Bran oil	[138]
**429**	4,12-Tritriacontadiene	Hulls	[73]
**430**	Oryzatriacontolide	Hulls	[139]
**431**	Benzoic acid	Hulls	[73]
**432**	γ-Nonalactone	Straw	[140]
**433**	Dicyclohexylorizane	Hulls	[141]
**434**	4-(3-Formylheptyl)cyclo- hexyl 4-(cyclohexylcarbonyloxymethyl)cyclo-hexyl-carboxylate	Hulls	[137]
**435**	*cis*-12-Oxo-Phytodienoicacid	Whole plants	[142]
**436**	*β*-D-Xylopyranosyl-(2→1')-*O*-*α*-D-arabinopyranosyl-(2'→1'')-*O*-*α*-D-arabinopyranosyl-(2''→1''')-*O*-*α*-D-arabinopyranosyl-(2'''→1''')-*O*-*α*-D-arabinopyranosyide	Straw, Leaves	[64]
**437**	Oryzativol C	Roots	[143]
**438**	1-Phenyl-2-hydroxy-3,7-dimethyl-11-aldehydic-tetradecane-2-β-D-glucopyranoside	Hulls	[73]
**439**	(5*S*)-5-(Acetyloxy)-3-(1-methyl*ent*hyl)-2-cyclohexen-1-one=3-Isopropyl-5-acetoxycyclohexene-2-one-1	Leaves	[205, 212]

References

6. Park, M.-H.; Chung, I.-M.; Ahmad, A.; Kim, B.-H.; Hwang, S.-J. Growth inhibition of unicellular and colonial *Microcystis* strains (*Cyanophyceae*) by compounds isolated from rice (*Oryza sativa*) hulls. *Aquat. Bot.* **2009,** *90*, 309–314.
8. Kato-Noguchi, H.; Ota, K.; Ino, T. Release of momilactone A and B from rice plants into the rhizosphere and its bioactivities. *Allelopathy J.* **2008,** *22*, 321–328.
10. Zhang, Q.; Xu, Q.L.; Xia, X.M.; Dong, L.M.; Luo, B.; Liu, W.B.; Tan, J.W. A new phenylpropane-pimarane heterodimer and a new *ent*-kaurene diterpene from the husks of *Oryza sativa*. *Phytochem. Lett.* **2018,** *24*, 120–124.
12. Mitsuaki, T.; Akihiro, O.; Nobuki, S.; Chizuko, K.; Tadahiro, K.; Yoshio, K.; Norindo, T. Momilactone-C, a minor constituent of growth inhibitors in rice husk. *Chem. Lett.* **1976,** *5*, 1157–1158.
17. Horie, K.; Sakai, K.; Okugi, M.; Toshima, H.; Hasegawa, M. Ultraviolet-induced amides and casbene diterpenoids from rice leaves. *Phytochem. Lett.* **2016,** *15*, 57–62.
18. Koga, J.; Shimura, M.; Oshima, K.; Ogawa, N.; Yamauchi, T.; Ogasawara, N. Phytocassanes A, B, C and D, novel diterpene phytoalexins from rice, *Oryza sativa* L. *Tetrahedron* **1995,** *51*, 7907–7918.
19. Koga, J.; Ogawa, N.; Yamauchi, T.; Kikuchi, M.; Ogasawara, N.; Shimura, M. Functional moiety for the antifungal activity of phytocassane E, a diterpene phytoalexin from rice. *Phytochemistry* **1997,** *44*, 249–253.
20. Horie, K.; Inoue, Y.; Sakai, M.; Yao, Q.; Tanimoto, Y.; Koga, J.; Toshima, H.; Hasegawa, M. Identification of UV-induced diterpenes including a new diterpene phytoalexin, phytocassane F, from rice leaves by complementary GC/MS and LC/MS approaches. *J. Agric. Food Chem.* **2015,** *63*, 4050–4059.
21. Kono, Y.; Kojima, A.; Nagai, R.; Watanabe, M.; Kawashima, T.; Onizawa, T.; Teraoka, T.; Watanab, M.; Koshino, H.; Uzawa, J., et al. Antibacterial diterpenes and their fatty acid conjugates from rice leaves. *Phytochemistry* **2004,** *65*, 1291–1298.
22. Akatsuka, T.; Kodama, O.; Sekido, H.; Kono, Y.; Takeuchi, S. Novel phytoalexins (oryzalexins A, B and C) isolated from rice blast leaves infected with *Pyricularia oryzae*. Part I: isolation, characterization and biological activities of oryzalexins. *Agric. Biol. Chem.* **1985,** *49*, 1689–1694.
23. Cho, J.-G.; Cha, B.-J.; Min Lee, S.; Shrestha, S.; Jeong, R.-H.; Sung Lee, D.; Kim, Y.-C.; Lee, D.-G.; Kang, H.-C.; Kim, J., et al. Diterpenes from the roots of *Oryza sativa* L. and their inhibition activity on NO production in LPS-stimulated RAW264.7 macrophages. *Chem. Biodivers.* **2015,** *12*, 1356–1364.
24. Cartwright, D.W.; Langcake, P.; Pryce, R.J.; Leworthy, D.P.; Ride, J.P. Isolation and characterization of two phytoalexins from rice as momilactones A and B. *Phytochemistry* **1981,** *20*, 535–537.
25. Kato, T.; Kabuto, C.; Sasaki, N.; Tsunagawa, M.; Aizawa, H.; Fujita, K.; Kato, Y.; Kitahara, Y.; Takahashi, N. Momilactones, growth inhibitors from rice, *Oryza sativa* L. *Tetrahedron Lett.* **1973,** *14*, 3861–3864.
26. Chung, I.M.; Kim, J.T.; Kim, S.-H. Evaluation of allelopathic potential and quantification of momilactone A,B from rice hull extracts and assessment of inhibitory bioactivity on paddy field weeds. *J. Agric. Food Chem.* **2006,** *54*, 2527–2536.
27. Ramazani, E.; Akaberi, M.; Emami, S.A.; Tayarani-Najaran, Z. Biological and pharmacological effects of γ-oryzanol: an updated review of the molecular mechanisms. *Curr. Pharm. Design.* **2021,** *27*, 2299–2316.
28. Chumpolsri, W.; Wijit, N.; Boontakham, P.; Nimmanpipug, P.; Sookwong, P.; Luangkamin, S.; Wongpornchai, S. Variation of terpenoid flavor odorants in bran of some black and white rice varieties analyzed by GC×GC-MS. *J. Food Nutr. Res.* **2015,** *3*, 114–120.
29. Lee, G.W.; Chung, M.-S.; Kang, M.; Chung, B.Y.; Lee, S. Direct suppression of a rice bacterial blight (*Xanthomonas oryzae* pv. *oryzae*) by monoterpene (*S*)-limonene. *Protoplasma* **2016,** *253*, 683–690.
30. Obara, N.; Hasegawa, M.; Kodama, O. Induced volatiles in elicitor-treated and rice blast fungus-inoculated rice leaves. *Biosci. Biotech. Bioch.* **2002,** *66*, 2549–2559.
31. Taniguchi, S.; Hosokawa-Shinonaga, Y.; Tamaoki, D.; Yamada, S.; Akimitsu, K.; Gomi, K. Jasmonate induction of the monoterpene linalool confers resistance to rice bacterial blight and its biosynthesis is regulated by JAZ protein in rice. *Plant Cell Environ.* **2014,** *37*, 451–461.
32. Kiyama, H.; Matsunaga, A.; Suzuki, G.; Gomi, K. Monoterpene geraniol produced by rice terpene synthase 21 suppresses the expression of cell-division related genes in the rice bacterial pathogen, *Xanthomonas oryzae* pv. *oryzae*. *Physiol. Mol. Plant P.* **2021,** *115*, 101673.
33. Lee, H.; Lee, G.; Kim, Y.; Ahn, H.; Lee, K.-G. Analysis of volatile compounds and antioxidant activity in rice extracts (*Oryza sativa* L.) extracted by various conditions. *Inst. Food Sci. Tech.* **2022,** *57*, 5289–5296.
34. Yoshitomi, K.; Taniguchi, S.; Tanaka, K.; Uji, Y.; Akimitsu, K.; Gomi, K. Rice terpene synthase 24 (OsTPS24) encodes a jasmonate-responsive monoterpene synthase that produces an antibacterial γ-terpinene against rice pathogen. *J. Plant Physiol.* **2016,** *191*, 120–126.
35. Concepcion, J.C.T.; Ouk, S.; Riedel, A.; Calingacion, M.; Zhao, D.; Ouk, M.; Garson, M.J.; Fitzgerald, M.A. Quality evaluation, fatty acid analysis and untargeted profiling of volatiles in Cambodian rice. *Food Chem.* **2018,** *240*, 1014–1021.
36. Kiryu, M.; Hamanaka, M.; Yoshitomi, K.; Mochizuki, S.; Akimitsu, K.; Gomi, K. Rice terpene synthase 18 (OsTPS18) encodes a sesquiterpene synthase that produces an antibacterial (*E*)-nerolidol against a bacterial pathogen of rice. *J. Gen. Plant Pathol.* **2018,** *84*, 221–229.
37. Chung, I.M.; Ali, M.; Hahn, S.J.; Siddiqui, N.A.; Lim, Y.H.; Ahmad, A. Chemical constituents from the hulls of *Oryza sativa* with cytotoxic activity. *Chem. Nat. Compd.* **2005,** *41*, 182–189.

38. Sridharan, A.; Thankappan, S.; Karthikeyan, G.; Uthandi, S. Comprehensive profiling of the VOCs of *Trichoderma longibrachiatum* EF5 while interacting with *Sclerotium rolfsii* and *Macrophomina phaseolina*. *Microbiol. Res.* **2020,** *236*, 126436.
39. Xiao, Y.H.; Wang, Q.J.; Erb, M.; Turlings, T.C.; Ge, L.M.; Hu, L.F.; Li, J.; Han, X.; Zhang, T.; Lu, J. Specific herbivore-induced volatiles defend plants and determine insect community composition in the field. *Ecol. Lett.* **2012,** *15*, 1130–1139.
40. Inoue, Y.; Sakai, M.; Yao, Q.; Tanimoto, Y.; Toshima, H.; Hasegawa, M. Identification of a novel casbane-type diterpene phytoalexin, *ent*-10-oxodepressin, from rice leaves. *Biosci. Biotech. Bioch.* **2013,** *77*, 760–765.
41. Li, G.; Xu, Q.L.; He, C.M.; Zeng, L.; Wang, H.F. Two new anti-fungal diterpenoids from the husks of *Oryza sativa*. *Phytochem. Lett.* **2014,** *10*, 309–312.
42. Gu, C.Z.; Xia, X.M.; Lv, J.; Tan, J.W.; Baerson, S.R.; Pan, Z.Q.; Song, Y.Y.; Zeng, R.S. Diterpenoids with herbicidal and antifungal activities from hulls of rice (*Oryza sativa*). *Fitoterapia* **2019,** *136*, 104183.
43. Kodama, O.; Li, W.X.; Tamogami, S.; Akatsuka, T. Oryzalexin S, a novel stemarane-type diterpene rice phytoalexin. *Biosci. Biotech. Bioch.* **1992,** *56*, 1002–1003.
44. Kato, H.; Kodama, O.; Akatsuka, T. Oryzalexin E, a diterpene phytoalexin from UV-irradiated rice leaves. *Phytochemistry* **1993,** *33*, 79–81.
45. Nemoto, T.; Cho, E.-M.; Okada, A.; Okada, K.; Otomo, K.; Kanno, Y.; Toyomasu, T.; Mitsuhashi, W.; Sassa, T.; Minami, E. Stemar-13-ene synthase, a diterpene cyclase involved in the biosynthesis of the phytoalexin oryzalexin S in rice. *FEBS Lett.* **2004,** *571*, 182–186.
46. Kurogochi, S.; Murofushi, N.; Ota, Y.; Takahashi, N. Identification of gibberellins in the rice plant and quantitative changes of gibberellin A_{19} throughout its life cycle. *Planta* **1979,** *146*, 185–191.
47. Shimura, K.; Okada, A.; Okada, K.; Jikumaru, Y.; Ko, K.-W.; Toyomasu, T.; Sassa, T.; Hasegawa, M.; Kodama, O.; Shibuya, N. Identification of a biosynthetic gene cluster in rice for momilactones. *J. Biol. Chem.* **2007,** *282*, 34013–34018.
48. Wu, Y.S.; Wang, Q.; Hillwig, M.L.; Peters, R.J. Picking sides: distinct roles for CYP76M6 and CYP76M8 in rice oryzalexin biosynthesis. *Biochem. J.* **2013,** *454*, 209–216.
49. Kato, H.; Kodama, O.; Akatsuka, T. Oryzalexin F, a diterpene phytoalexin from UV-irradiated rice leaves. *Phytochemistry* **1994,** *36*, 299–301.
50. Kato-Noguchi, H.; Ino, T. Concentration and release level of momilactone B in the seedlings of eight rice cultivars. *J. Plant Physiol.* **2005,** *162*, 965–969.
53. Verardo, V.; Gómez-Caravaca, A.M.; Marconi, E.; Segura-Carretero, A.; Garrido-Frenich, A.; Fernández-Gutiérrez, A. Determination of lipophilic and hydrophilic bioactive compounds in raw and parboiled rice bran. *RSC Adv.* **2016,** *6*, 50786–50796.
54. Akihisa, T.; Yasukawa, K.; Yamaura, M.; Ukiya, M.; Kimura, Y.; Shimizu, N.; Arai, K. Triterpene alcohol and sterol ferulates from rice bran and their anti-inflammatory effects. *J. Agric. Food Chem.* **2000,** *48*, 2313–2319.
55. Suttiarporn, P.; Chumpolsri, W.; Mahatheeranont, S.; Luangkamin, S.; Teepsawang, S.; Leardkamolkarn, V. Structures of phytosterols and triterpenoids with potential anti-cancer activity in bran of black non-glutinous rice. *Nutrients* **2015,** *7*, 1672–1687.
56. Fang, N.B.; Yu, S.G.; Badger, T.M. Characterization of triterpene alcohol and sterol ferulates in rice bran using LC-MS/MS. *J. Agric. Food Chem.* **2003,** *51*, 3260–3267.
57. Oka, T.; Fujimoto, M.; Nagasaka, R.; Ushio, H.; Hori, M.; Ozaki, H. Cycloartenyl ferulate, a component of rice bran oil-derived γ-oryzanol, attenuates mast cell degranulation. *Phytomedicine* **2010,** *17*, 152–156.
58. Chung, I.M.; Ali, M.; Ahmad, A.; Lim, J.D.; Yu, C.Y.; Kim, J.S. Chemical constituents of rice (*Oryza sativa*) hulls and their herbicidal activity against duckweed (*Lemna paucicostata* Hegelm 381). *Phytochem. Analysis* **2006,** *17*, 36–45.
59. Sabir, A.; Rafi, M.; Darusman, L.K. Discrimination of red and white rice bran from Indonesia using HPLC fingerprint analysis combined with chemometrics. *Food Chem.* **2017,** *221*, 1717–1722.
60. Liu, C.; Xi, X.J.; Liu, Y.Y.; Lu, Y.Z.; Che, F.F.; Gu, Y.X.; Yu, Y.C.; Li, H.; Liu, J.G.; Wei, Y. Isolation of four major compounds of γ-oryzanol from rice bran oil by ionic liquids modified high-speed countercurrent chromatography and antimicrobial activity and neuroprotective effect of cycloartenyl ferulate *in vitro*. *Chromatographia* **2021,** *84*, 635–644.
61. Luo, H.F.; Li, Q.L.; Yu, S.G.; Badger, T.M.; Fang, N.B. Cytotoxic hydroxylated triterpene alcohol ferulates from rice bran. *J. Nat. Prod.* **2005,** *68*, 94–97.
62. Zhang, W.H.; Zhong, H.M.; Che, C.T. Cycloartanes from the red alga *Galaxaura* sp. *J. Asian Nat. Prod. Res.* **2005,** *7*, 59–65.
63. Shu, X.L.; Frank, T.; Shu, Q.Y.; Engel, K.-H. Metabolite profiling of germinating rice seeds. *J. Agric. Food Chem.* **2008,** *56*, 11612–11620.
64. Chung, I.M.; Kwon, C.; An, Y.; Ali, M.; Lee, H.; Lim, J.-D.; Kim, S.; Yang, Y.J.; Kim, S.-H.; Ahmad, A. Characterization of new polyphenolic glycosidic constituents and evaluation of cytotoxicity on a macrophage cell line and allelopathic activities of *Oryza sativa*. *Molecules* **2018,** *23*, 1933.
65. Chung, I.M.; Ali, M.; Ahmad, A. A and B from rice hulls of *Oryza sativa*. *Indian J. Chem.* **2007,** *3*, 516–522.
66. Norvienyeku, J.; Lin, L.L.; Waheed, A.; Chen, X.M.; Bao, J.D.; Aliyu, S.R.; Lin, L.Y.; Shabbir, A.; Batool, W.; Zhong, Z.H., et al. Bayogenin 3-*O*-cellobioside confers non-cultivar-specific defence against the rice blast fungus *Pyricularia oryzae*. *Plant Biotechnol. J.* **2021,** *19*, 589–601.
67. Okahara, F.; Suzuki, J.; Hashizume, K.; Osaki, N.; Shimotoyodome, A. Triterpene alcohols and sterols from rice bran reduce postprandial hyperglycemia in rodents and humans. *Mol. Nutr. Food Res.* **2016,** *60*, 1521–1531.
68. Sen, S.; Chakraborty, R.; Kalita, P. Rice-not just a staple food: a comprehensive review on its phytochemicals and therapeutic potential. *Trends Food Sci. Tech.* **2020,** *97*, 265–285.

69. Ali, M.; Ahmad, A.; Sultana, S.; Mir, S.R. Chemical constituents from the seed husks of *Oryza sativa* L. *Nat. Prod. Res.* **2022,** *36*, 5530–5538.
70. Jung, Y.J.; Park, J.-H.; Shrestha, S.; Song, M.-C.; Cho, S.; Lee, C.-H.; Han, D.; Baek, N.-I. Phytosterols from the rice (*Oryza sativa*) bran. *J. Appl. Biol. Chem.* **2014,** *57*, 175–178.
71. Ohnishi, M.; Fujino, Y. Novel glycolipids; cellobiosylsterol and cellotriosylsterol in rice bran. *Agric. Biol. Chem.* **1978,** *42*, 2423–2425.
72. Ohnishi, M.; Fujino, Y. Structural study on new sterylglycosides in rice bran: cellotetraosylsitosterol and cellopentaosylsitosterol. *Agric. Biol. Chem.* **1980,** *44*, 333–338.
73. Chung, I.M.; Ali, M.; Ahmad, A.; Chun, S.-C.; Kim, J.-T.; Sultana, S.; Kim, J.-S.; Min, S.-K.; Seo, B.-R. Steroidal constituents of rice (*Oryza sativa*) hulls with algicidal and herbicidal activity against blue–green algae and duckweed. *Phytochem. Analysis* **2007,** *18*, 133–145.
74. Luang-In, V.; Yotchaisarn, M.; Somboonwatthanakul, I.; Deeseenthum, S. Bioactivities of organic riceberry broken rice and crude riceberry rice oil. *J. Pharm. Anal.* **2018,** *42*.
77. Ding, C.; Liu, Q.; Li, P.; Pei, Y.S.; Tao, T.T.; Wang, Y.; Yan, W.; Yang, G.F.; Shao, X.L. Distribution and quantitative analysis of phenolic compounds in fractions of Japonica and Indica rice. *Food Chem.* **2019,** *274*, 384–391.
78. Wang, W.; Guo, J.; Zhang, J.N.; Peng, J.; Liu, T.X.; Xin, Z.H. Isolation, identification and antioxidant activity of bound phenolic compounds present in rice bran. *Food Chem.* **2015,** *171*, 40–49.
79. Seal, A.N.; Haig, T.; Pratley, J.E. Evaluation of putative allelochemicals in rice root exudates for their role in the suppression of arrowhead root growth. *J. Chem. Ecol.* **2004,** *30*, 1663–1678.
80. Ti, H.H.; Li, Q.; Zhang, R.F.; Zhang, M.W.; Deng, Y.Y.; Wei, Z.C.; Chi, J.W.; Zhang, Y. Free and bound phenolic profiles and antioxidant activity of milled fractions of different indica rice varieties cultivated in southern China. *Food Chem.* **2014,** *159*, 166–174.
81. Rosado, M.J.; Rencoret, J.; Marques, G.; Gutiérrez, A.; Del Río, J.C. Structural characteristics of the guaiacyl-rich lignins from rice (*Oryza sativa* L.) husks and straw. *Front. Plant Sci.* **2021,** *12*, 640475.
82. Yang, Z.G.; Nakabayashi, R.; Okazaki, Y.; Mori, T.; Takamatsu, S.; Kitanaka, S.; Kikuchi, J.; Saito, K. Toward better annotation in plant metabolomics: isolation and structure elucidation of 36 specialized metabolites from *Oryza sativa* (rice) by using MS/MS and NMR analyses. *Metabolomics* **2014,** *10*, 543–555.
83. Zeng, Z.C.; Hu, X.T.; McClements, D.J.; Luo, S.J.; Liu, C.M.; Gong, E.; Huang, K. Hydrothermal stability of phenolic extracts of brown rice. *Food Chem.* **2019,** *271*, 114–121.
84. Zaupa, M.; Calani, L.; Del Rio, D.; Brighenti, F.; Pellegrini, N. Characterization of total antioxidant capacity and (poly) phenolic compounds of differently pigmented rice varieties and their changes during domestic cooking. *Food Chem.* **2015,** *187*, 338–347.
85. Han, S.J.; Ryu, S.N.; Kang, S.S. A new 2-arylbenzofuran with antioxidant activity from the black colored rice (*Oryza sativa* L.) bran. *Chem. Pharm. Bull.* **2004,** *52*, 1365–1366.
86. Himeno, N.; Saburi, W.; Wakuta, S.; Takeda, R.; Matsuura, H.; Nabeta, K.; Sansenya, S.; Cairns, J.R.K.; Mori, H.; Imai, R. Identification of rice β-glucosidase with high hydrolytic activity towards salicylic acid β-D-glucoside. *Biosci. Biotech. Bioch.* **2013,** *77*, 934–939.
87. Tanaka, J.; Nakanishi, T.; Shimoda, H.; Nakamura, S.; Tsuruma, K.; Shimazawa, M.; Matsuda, H.; Yoshikawa, M.; Hara, H. Purple rice extract and its constituents suppress endoplasmic reticulum stress-induced retinal damage *in vitro* and *in vivo*. *Life Sci.* **2013,** *92*, 17–25.
90. Deng, G.F.; Xu, X.R.; Zhang, Y.; Li, D.; Gan, R.Y.; Li, H.B. Phenolic compounds and bioactivities of pigmented rice. Crit. Rev. Food Sci. 2013, 53, 296–306.
92. Zhang, F.; Yang, L.M.; Huang, W.X.; Luo, X.D.; Xie, J.K.; Hu, B.L.; Chen, Y.L. Flavonoid metabolic profiles and gene mapping of rice (*Oryza sativa* L.) purple gradient grain hulls. *Rice* **2022,** *15*, 43.
93. Irakli, M.N.; Samanidou, V.F.; Biliaderis, C.G.; Papadoyannis, I.N. Simultaneous determination of phenolic acids and flavonoids in rice using solid-phase extraction and RP-HPLC with photodiode array detection. *J. Sep. Sci.* **2012,** *35*, 1603–1611.
94. Yu, X.T.; Yang, T.; Qi, Q.Q.; Du, Y.M.; Shi, J.; Liu, X.M.; Liu, Y.H.; Zhang, H.B.; Zhang, Z.F.; Yan, N. Comparison of the contents of phenolic compounds including flavonoids and antioxidant activity of rice (*Oryza sativa*) and Chinese wild rice (*Zizania latifolia*). *Food Chem.* **2021,** *344*, 128600.
95. Shimizu, T.; Lin, F.Q.; Hasegawa, M.; Okada, K.; Nojiri, H.; Yamane, H. Purification and identification of naringenin 7-*O*-methyltransferase, a key enzyme in biosynthesis of flavonoid phytoalexin sakuranetin in rice. *J. Biol. Chem.* **2012,** *287*, 19315–19325.
96. Chung, I.M.; Park, S.-K.; Ali, M.; Prabakaran, M.; Oh, Y.-T.; Kim, S.-H.; Siddiqui, N.A.; Ahmad, A. Flavonoid glycosides from leaves and straw of *Oryza sativa* and their effects of cytotoxicity on a macrophage cell line and allelopathic on weed germination. *Saudi. Pharm. J.* **2018,** *26*, 375–387.
97. Kodama, O.; Miyakawa, J.; Akatsuka, T.; Kiyosawa, S. Sakuranetin, a flavanone phytoalexin from ultraviolet-irradiated rice leaves. *Phytochemistry* **1992,** *31*, 3807–3809.
98. Katsumata, S.; Hamana, K.; Horie, K.; Toshima, H.; Hasegawa, M. Identification of sternbin and naringenin as detoxified metabolites from the rice flavanone phytoalexin sakuranetin by *Pyricularia oryzae*. *Chem. Biodivers.* **2017,** *14*, e1600240.
99. Katsumata, S.; Toshima, H.; Hasegawa, M. Xylosylated detoxification of the rice flavonoid phytoalexin sakuranetin by the rice sheath blight fungus *Rhizoctonia solani*. *Molecules* **2018,** *23*, 276.

100. Jan, R.; Khan, M.; Asaf, S.; Lubna; Asif, S.; Kim, K.-M. Bioactivity and therapeutic potential of kaempferol and quercetin: new insights for plant and human health. *Plants* **2022,** *11*, 2623.
101. Kim, B.; Woo, S.; Kim, M.-J.; Kwon, S.-W.; Lee, J.; Sung, S.H.; Koh, H.-J. Identification and quantification of flavonoids in yellow grain mutant of rice (*Oryza sativa* L.). *Food Chem.* **2018,** *241*, 154–162.
102. Jeong, R.-H.; Lee, D.-Y.; Cho, J.-G.; Lee, S.-M.; Kang, H.-C.; Seo, W.-D.; Kang, H.-W.; Kim, J.-Y.; Baek, N.-I. A new flavonolignan from the aerial parts of *Oryza sativa* L. inhibits nitric oxide production in RAW 264.7 macrophage cells. *J. Korean Soc. Appl. Biol. Chem.* **2011,** *54*, 865–870.
103. Kim, C.; Kikuchi, S.; Kim, Y.; Park, S.; Yoon, U.; Lee, G.; Choi, J.; Kim, Y.; Park, S. Computational identification of seed-specific transcription factors involved in anthocyanin production in black rice. *Biochip J.* **2010,** *4*, 247–255.
104. Hou, Z.H.; Qin, P.Y.; Zhang, Y.; Cui, S.H.; Ren, G.X. Identification of anthocyanins isolated from black rice (*Oryza sativa* L.) and their degradation kinetics. *Food Res. Int.* **2013,** *50*, 691–697.
105. Chen, X.Q.; Nagao, N.; Itani, T.; Irifune, K. Anti-oxidative analysis, and identification and quantification of anthocyanin pigments in different coloured rice. *Food Chem.* **2012,** *135*, 2783–2788.
106. Hao, J.; Zhu, H.; Zhang, Z.Q.; Yang, S.L.; Li, H.R. Identification of anthocyanins in black rice (*Oryza sativa* L.) by UPLC/Q-TOF-MS and their *in vitro* and *in vivo* antioxidant activities. *J. Cereal Sci.* **2015,** *64*, 92–99.
107. Tamura, S.; Yan, K.; Shimoda, H.; Murakami, N. Anthocyanins from *Oryza sativa* L. subsp. *indica*. *Biochem. Syst. Ecol.* **2010,** *38*, 438–440.
108. Besson, E.; Dellamonica, G.; Chopin, J.; Markham, K.R.; Kim, M.; Koh, H.-S.; Fukami, H. C-glycosylflavones from *Oryza sativa*. *Phytochemistry* **1985,** *24*, 1061–1064.
109. Cho, J.-G.; Song, N.Y.; Nam, T.-G.; Shrestha, S.; Park, H.-J.; Lyu, H.-N.; Kim, D.-O.; Lee, G.; Woo, Y.-M.; Jeong, T.-S., et al. Flavonoids from the grains of C1/R-S transgenic rice, the transgenic *Oryza sativa* spp. *japonica*, and their radical scavenging activities. *J. Agric. Food Chem.* **2013,** *61*, 10354–10359.
110. Yang, Z.G.; Nakabayashi, R.; Mori, T.; Takamatsu, S.; Kitanaka, S.; Saito, K. Metabolome analysis of *Oryza sativa* (rice) using liquid chromatography-mass spectrometry for characterizing organ specificity of flavonoids with anti-inflammatory and antioxidant activity. *Chem. Pharm. Bull.* **2016,** *64*, 952–956.
111. Ajitha, M.J.; Mohanlal, S.; Suresh, C.H.; Jayalekshmy, A. DPPH radical scavenging activity of tricin and its conjugates isolated from "njavara" rice bran: a density functional theory study. *J. Agric. Food Chem.* **2012,** *60*, 3693–3699.
112. Mohanlal, S.; Parvathy, R.; Shalini, V.; Helen, A.; Jayalekshmy, A. Isolation, characterization and quantification of tricin and flavonolignans in the medicinal rice njavara (*Oryza sativa* L.), as compared to staple varieties. *Plant Food Hum. Nutr.* **2011,** *66*, 91–96.
113. Qureshi, A.A.; Mo, H.; Packer, L.; Peterson, D.M. Isolation and identification of novel tocotrienols from rice bran with hypocholesterolemic, antioxidant, and antitumor properties. *J. Agric. Food Chem.* **2000,** *48*, 3130–3140.
114. Sookwong, P.; Murata, K.; Nakagawa, K.; Shibata, A.; Kimura, T.; Yamaguchi, M.; Kojima, Y.; Miyazawa, T. Cross-fertilization for enhancing tocotrienol biosynthesis in rice plants and QTL analysis of their F2 progenies. *J. Agric. Food Chem.* **2009,** *57*, 4620–4625.
115. Jeong, I.-M.; Lim, Y.-H.; Ali, M.; Sultana, S.; Ahmad, A. Novel anthracene derivatives isolated from rice hulls of *Oryza sativa* and their growth inhibitory activity of radish seed. *B. Korean Chem. Soc.* **2006,** *27*, 995–1000.
116. Chung, I.M.; Park, H.Y.; Chun, S.C.; Kim, J.J.; Ahmad, A. New glycosidic and other constituents from hulls of *Oryza sativa*. *Chem. Nat. Compd.* **2007,** *43*, 417–421.
117. Chen, M.-H.; McClung, A.M.; Bergman, C.J. Phenolic content, anthocyanins and antiradical capacity of diverse purple bran rice genotypes as compared to other bran colors. *J. Cereal Sci.* **2017,** *77*, 110–119.
119. Aboshi, T.; Iitsuka, C.; Galis, I.; Teraishi, M.; Kamo, M.; Nishimura, A.; Ishihara, A.; Mori, N.; Murayama, T. Isopentylamine is a novel defence compound induced by insect feeding in rice. *Plant Cell Environ.* **2021,** *44*, 247–256.
120. Morimoto, N.; Ueno, K.; Teraishi, M.; Okumoto, Y.; Mori, N.; Ishihara, A. Induced phenylamide accumulation in response to pathogen infection and hormone treatment in rice (*Oryza sativa*). *Biosci. Biotech. Bioch.* **2018,** *82*, 407–416.
121. Park, H.L.; Yoo, Y.; Hahn, T.-R.; Bhoo, S.H.; Lee, S.-W.; Cho, M.H. Antimicrobial activity of UV-induced phenylamides from rice leaves. *Molecules* **2014,** *19*, 18139–18151.
122. Wang, W.W.; Yu, Z.X.; Meng, J.P.; Zhou, P.Y.; Luo, T.; Zhang, J.; Wu, J.; Lou, Y.G. Rice phenolamindes reduce the survival of female adults of the white-backed planthopper *Sogatella furcifera*. *Sci. Rep.* **2020,** *10*, 5778.
123. Alamgir, K.M.; Hojo, Y.; Christeller, J.T.; Fukumoto, K.; Isshiki, R.; Shinya, T.; Baldwin, I.T.; Galis, I. Systematic analysis of rice (*Oryza sativa*) metabolic responses to herbivory. *Plant Cell Environ.* **2016,** *39*, 453–466.
124. Buttery, R.G.; Ling, L.C.; Juliano, B.O.; Turnbaugh, J.G. Cooked rice aroma and 2-acetyl-1-pyrroline. *J. Agric. Food Chem.* **1983,** *31*, 823–826.
125. Kinashi, H.; Suzuki, Y.; Takeuchi, S.; Kawarada, A. Possible metabolic intermediates from IAA to β-acid in rice bran. *Agric. Biol. Chem.* **1976,** *40*, 2465–2470.
126. Suzuki, Y.; Kinashi, H.; Takeuchi, S.; Kawarada, A. (+)-5-Hydroxy-dioxindole-3-acetic acid, a synergist from rice bran of auxin-induced ethylene production in plant tissue. *Phytochemistry* **1977,** *16*, 635–637.
127. Yamamoto, Y.; Kamiya, N.; Morinaka, Y.; Matsuoka, M.; Sazuka, T. Auxin biosynthesis by the YUCCA genes in rice. *Plant Physiol.* **2007,** *143*, 1362–1371.

128. Ishihara, A.; Hashimoto, Y.; Miyagawa, H.; Wakasa, K. Induction of serotonin accumulation by feeding of rice striped stem borer in rice leaves. *Plant Signal. Behav.* **2008,** *3*, 714–716.
129. Nakano, H.; Ono, H.; Kaji, R.; Sakai, M.; Doi, S.; Kosemura, S. Oryzadiamines A and B, alkaloids from *Oryza sativa* with yellow grain. *Tetrahedron Lett.* **2020,** *61*, 151519.
131. Nakano, H.; Kosemura, S.; Suzuki, T.; Hirose, K.; Kaji, R.; Sakai, M. Oryzamutaic acid A, a novel yellow pigment from an *Oryza sativa* mutant with yellow endosperm. *Tetrahedron Lett.* **2009,** *50*, 2003−2005.
132. Nakano, H.; Kosemura, S.; Yoshida, M.; Suzuki, T.; Iwaura, R.; Kaji, R.; Sakai, M.; Hirose, K. Oryzamutaic acids B–G, new alkaloids from an *Oryza sativa* mutant with yellow endosperm. *Tetrahedron Lett.* **2010,** *51*, 49–53.
133. Nakano, H.; Kosemura, S.; Yoshida, M.; Iwaura, R.; Suzuki, T.; Kaji, R.; Sakai, M. Oryzamutaic acids H–J, new alkaloids from an *Oryza sativa* mutant with yellow endosperm. *Tetrahedron Lett.* **2010,** *51*, 4953–4956.
135. Tanaka, K.; Taniguchi, S.; Tamaoki, D.; Yoshitomi, K.; Akimitsu, K.; Gomi, K. Multiple roles of plant volatiles in jasmonate-induced defense response in rice. *Plant Signal. Behav.* **2014,** *9*, e29247.
136. Fujino, Y.; Ohnishi, M. Constituents of ceramide and ceramide monohexoside in rice bran. *Chem. Phys. Lipids* **1976,** *17*, 275–289.
137. Chung, I.M.; Ali, M.; Chun, S.-C.; Jin, C.W.; Cho, D.H.; Hong, S.-B.; Ahmad, A. New aliphatic alcohol and ester constituents from rice hulls of *Oryza sativa*. *Chinese J. Chem.* **2007,** *25*, 843–848.
138. Ahmad, A.; Yoon, J.; Chung, I. Chemical constituents from the rice straw of *Oryza sativa*. *Asian J. Chem.* **2013,** *25*, 9872–9874.
139. Chung, I.M.; Ali, M.; Chun, S.-C.; Lee, O.-K.; Ahmad, A. Sativalanosteronyl glycoside and oryzatriacontolide constituents from the Hulls of *Oryza sativa*. *Asian J. Chem.* **2007,** *19*, 1535.
140. Miyazawa, M.; Nagai, S.; Oshima, T. Volatile components of the straw of *Oryza sativa* L. *J. Oleo Sci.* **2008,** *57*, 139–143.
141. Chung, I.M.; Ali, M.; Ahmad, A. Dicyclohexanyl orizane constituent from the hulls of *Oryza sativa* and its inhibitory activity. *Asian J. Chem.* **2005,** *17*, 2616.
142. Guo, H.M.; Li, H.C.; Zhou, S.R.; Xue, H.W.; Miao, X.X. *cis*-12-Oxo-phytodienoic acid stimulates rice defense response to a piercing-sucking insect. *Mol. Plant* **2014,** *7*, 1683–1692.
143. Lee, T.K.; Lee, D.; Yu, J.S.; Jo, M.S.; Baek, S.C.; Shin, M.-S.; Ko, Y.-J.; Kang, K.S.; Kim, K.H. Biological evaluation of a new lignan from the roots of rice (*Oryza sativa*). *Chem. Biodivers.* **2018,** *15*, e1800333.
146. Zhan, Z.H.; Matsuo, A.; Oku, Y.; Tebayashi, S.-i.; Kim, C.-S. Studies on the probing stimulants for the white-backed planthopper, *Sogatella furcifera* (Homoptera: Delphacidae) in rice plant (*Oryza sativa* L.). *Biosci. Biotech. Bioch.* **2016,** *80*, 2285–2290.
170. Takeda, Y.; Koshiba, T.; Tobimatsu, Y.; Suzuki, S.; Murakami, S.; Yamamura, M.; Rahman, M.M.; Takano, T.; Hattori, T.; Sakamoto, M. Regulation of CONIFERALDEHYDE 5-HYDROXYLASE expression to modulate cell wall lignin structure in rice. *Planta* **2017,** *246*, 337–349.
178. Watanabe, M.; Kono, Y.; Watanabe, M.; Uzawa, J.; Teraoka, T.; Hosokawa, D.; Suzuki, Y.; Sakurai, A.; Teraguchi, M. Structures of oryzalic acid B and three related compounds, a group of novel antibacterial diterpenes, isolated from leaves of a bacterial leaf blight-resistant cultivar of rice. *Biosci. Biotech. Bioch.* **1992,** *56*, 113–117.
179. Watanabe, M.; Sakai, Y.; Teraoka, T.; Abe, H.; Kono, Y.; Uzawa, J.; Kobayashi, K.; Suzuki, Y.; Sakurai, A. Novel C_{19}-kaurane type of diterpene (oryzalide A), a new antimicrobial compound isolated from healthy leaves of a bacterial leaf blight-resistant cultivar of rice plant. *Agric. Biol. Chem.* **1990,** *54*, 1103−1105.
180. Kono, Y.; Uzawa, J.; Kobayashi, K.; Suzuki, Y.; Uramoto, M.; Sakurai, A.; Watanabe, M.; Teraoka, T.; Hosokawa, D.; Watanabe, M., et al. Structures of oryzalides A and B, and oryzalic acid A, a group of novel antimicrobial diterpenes, isolated from healthy leaves of a bacterial leaf blight-resistant cultivar of rice plant. *Agric. Biol. Chem.* **1991,** *55*, 803−811.
184. Kono, Y.; Takeuchi, S.; Kodama, O.; Sekido, H.; Akatsuka, T. Novel phytoalexins (oryzalexins A, B and C) isolated from rice blast leaves infected with *Pyricularia oryzae*. Part II: structural studies of oryzalexins. *Agric. Biol. Chem.* **1985,** *49*, 1695–1701.
185. Sekido, H.; Endo, T.; Suga, T.; Kodama, O.; Akatsuka, T.; Kono, Y.; Takeuchi, S. Oryzalexin D (3, 7-dihydroxy-(+)-sandaracopimaradiene), a new phytoalexin isolated from blast-infected rice leaves. *J. Pestic. Sci.* **1986,** *11*, 369–372.
204. Thi, H.L.; Lin, C.H.; Smeda, R.J.; Leigh, N.D.; Wycoff, W.G.; Fritschi, F.B. Isolation and identification of an allelopathic phenylethylamine in rice. *Phytochemistry* **2014,** *108*, 109–121.
205. Kong, C.H.; Li, H.B.; Hu, F.; Xu, X.H.; Wang, P. Allelochemicals released by rice roots and residues in soil. *Plant Soil* **2006,** *288*, 47–56.
212. Kong, C.H.; Liang, W.J.; Xu, X.H.; Hu, F.; Wang, P.; Jiang, Y. Release and activity of allelochemicals from allelopathic rice seedlings. *J. Agric. Food Chem.* **2004,** *52*, 2861–2865.
